# Tunable GaAs_*x*_P_1–*x*_ Quantum-Dot Emission in Wurtzite GaP Nanowires

**DOI:** 10.1021/acsami.4c15343

**Published:** 2024-11-13

**Authors:** Robert Andrei Sorodoc, Paolo De Vincenzi, Akant Sagar Sharma, Giada Bucci, Mario Roggi, Enrico Mugnaioli, Lucia Sorba, Marta De Luca, Valentina Zannier

**Affiliations:** ‡ NEST Istituto Nanoscienze-CNR and Scuola Normale Superiore, Piazza S. Silvestro 12, 56127 Pisa, Italy; § Department of Physics, Sapienza University of Rome, P.le A. Moro 5, 00185 Rome, Italy; ⊥ Department of Earth Sciences, University of Pisa, Via S. Maria 53, 56126 Pisa, Italy

**Keywords:** nanowires, quantum dots, GaAsP, wurtzite GaP, microphotoluminescence

## Abstract

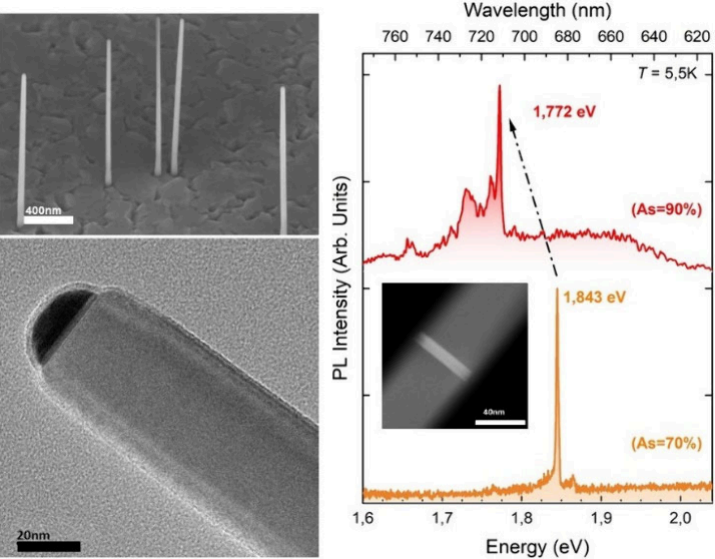

We present a fairly understudied material system suitable for the realization of tunable quantum-dot (QD) emission within the visible-to-near-infrared spectrum (in particular, in the 650–720 nm wavelength range). Specifically, crystal-pure wurtzite gallium phosphide (GaP) nanowires (NWs) are synthesized, incorporating single gallium arsenide phosphide (GaAs_*x*_P_1–*x*_) QDs of various compositions. Detailed growth procedures are outlined, accompanied by an analysis of the synthesis challenges encountered during the realization of these nanostructures and the strategies to solve them. Notably, a great degree of control over the shape and composition of the ternary alloy QD is achieved, enabling a well-defined confinement and the tunability of the emission wavelength. This is confirmed by low-temperature microphotoluminescence (μ-PL) investigation showing that the NW emission is dominated by a narrow peak whose energy shifts according to the As content of the QD: from ∼650 nm (As = 70%) to ∼720 nm (As = 90%). Moreover, a localized and efficient carrier recombination mechanism is found by single-NW μ-PL mapping, confirming that this emission arises from the QD. Finally, a power and temperature μ-PL study is presented and used to characterize the QD excitonic properties and the nature of the involved energy levels. Our findings underscore the potential for these QDs in NWs with tailored compositions to achieve the desired light emission characteristics, thereby advancing applications in quantum optics and nanophotonics.

## 1 Introduction

The emergence of bottom-up-grown nanowires (NWs) represents a significant step forward in the landscape of semiconductor technologies. Proofs of NW applications, including single-photon emitters and detectors,^1^ superconductive devices,^2,3^ and optoelectronic implementations,^4,5^ underline their pivotal role in advancing the field. Of particular interest are axially grown quantum-dot (QD) NWs, whose synthesis through compositional^6^ or crystal structural^7^ variation has garnered substantial attention. This interest comes from the unique opportunity afforded by the NW geometry to coherently grow highly lattice-mismatched materials, facilitated by the quasi-one-dimensional strain relaxation inherent in NWs,^8^ and to obtain crystal phases not achievable in other geometries, such as the wurtzite (WZ) in non-nitride III–V compounds.^9^

Nowadays, the quest for single-photon sources has assumed paramount importance, given their indispensable role in the development of quantum information technologies. Pure single-photon states, essential for tasks such as quantum key distribution and error minimization in quantum computing, can be obtained in several material systems, among which QDs are very attractive for their ease of integration in solid-state devices. Therefore, it is crucial to keep advancing QD growth techniques.^1,10^ While self-assembled planar QDs have undergone scrutiny and already found commercial utility due to their exceptional optical quality,^11^ the stochasticity inherent to the QD position and size in the Stransky–Krastanov technique is still an open challenge.^12^ Conversely, axial QD NWs grown using the vapor–liquid–solid technique exhibit remarkable precision and tunability in lateral and axial dimensions, as well as control of the QD chemical composition and positioning within the NW.^12^ Additional techniques such as selective area growth offer the possibility of obtaining waveguiding effects in the NWs, increasing the photon extraction efficiency.^12,13^

Material selection is a critical consideration, with gallium phosphide (GaP) assuming a prominent role within the III–V semiconductor class. The high transparency range (0.6–11 μm)^14^ and quasi-direct band gap in its WZ crystal structure in the range of 2.18–2.25 eV,^15−17^ coupled with minimal lattice mismatch with silicon, make GaP extremely interesting for the realization and integration of single-photon sources. Our research specifically focuses on the growth and tunability of WZ gallium phosphide arsenide (GaAs_*x*_P_1–*x*_) QDs within GaP NWs. The challenges posed by large lattice mismatches, which impede the realization of planar heterostructures combining GaAs and GaP, are circumvented in the NW geometry, opening new avenues for exploration and innovation and yielding promising results. Some works have explored the growth of QDs in GaAs/GaP NWs, but focusing only on the zinc blende (ZB) crystal structure and material combinations different from those in this work. Intense photoluminescence was observed from ZB GaAs QDs in GaAsP NWs, with antibunching phenomena up to 160 K, a full width at half-maximum (fwhm) of 1.2 meV (2 meV at 110 K), and an emission energy at 760 nm.^18^ Self-catalyzed GaAsP ZB NWs containing up to 50 GaAs QDs have been fabricated and show emission lines at 710 nm with fwhm values of <10 meV up to 140 K.^19^ It was shown that it is possible to incorporate GaAsP segments inside a single ZB GaP NW and obtain different QD emission energy by varying the As concentration.^20^ In the WZ crystal structure, the emission efficiency is expected to be higher due to the direct band gap.^16^ Importantly, defect-free WZ GaP NWs^21^ and all-WZ GaAs/GaP superlattice NWs^22^ can be obtained by Au-assisted chemical beam epitaxy, and control of the As/P flux ratio during the growth can allow for a high degree of tunability of the QD composition and hence light emission properties.^16^

In this work, we demonstrate the growth and morphology optimization of GaAs_*x*_P_1–*x*_ QDs, with As composition ranging between 10% and 90% embedded in defect-free WZ GaP NWs. We also show methods to eliminate NW tapering, as well as to enhance axial and radial GaP growth, which are crucial for having proper control over the NW diameter and length, in view of boosting the extraction rates by waveguiding the emitted photons. We prove, through microphotoluminescence (μ-PL) measurements, that control over the As content above 50% gives tunability over the QD emission wavelength, from 650 to 720 nm. We show that the emission arises from a localized area along the NW axis. The quantum-confined nanostructure efficiently captures the photogenerated carriers, resulting in a spectrally narrow and bright emission peak visible up 70 K (fwhm values of 2 meV at 5.5 K and <8 meV at 70 K), with negligible radiative recombination in the high-band-gap GaP barriers. Overall, this new NW material system shows the potential to act as a site-controlled and energy-controlled quantum light source at low temperatures.

## 2 Results and Discussion

**Figure 1:**
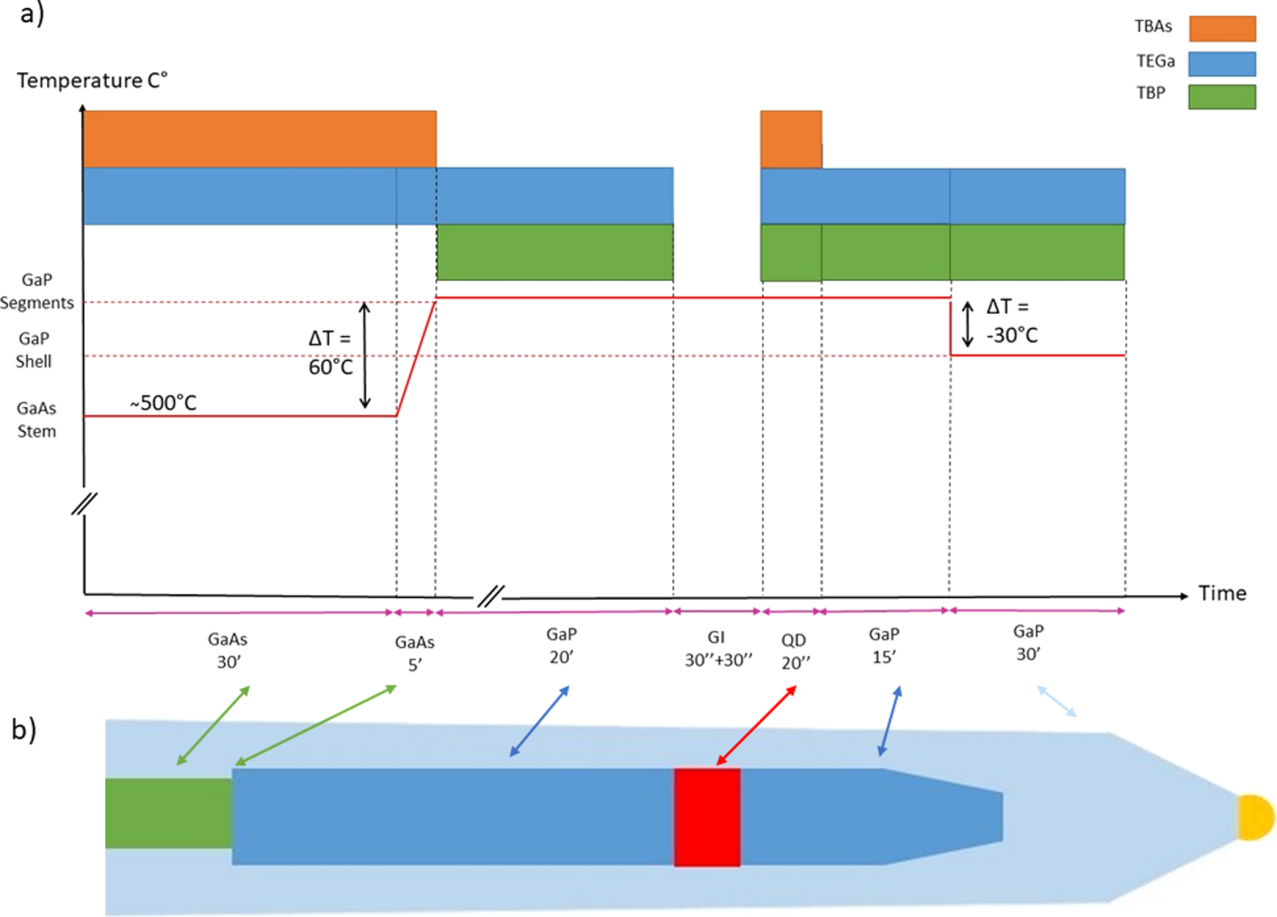
(a) Graphical representation (not to scale) of the growth procedure, with the metal–organic precursors used for each step indicated in different colors. The GI before the QD growth is used to prepare different Group V precursor pressures to tune the QD composition. (b) Schematics (not to scale) of the resulting nanostructure at the end of the entire growth process.

The growth sequence illustrated in Figure 1a is used to grow QD NWs, such as the one depicted in Figure 1b. It started with the growth for 30 min of a GaAs NW stem on a GaAs(111)B substrate at 500 °C using fixed triethylgallium (TEGa) and *tert*-butylarsine (TBAs) precursor line pressures of 0.7 and 0.7 Torr, respectively, resulting in NWs with a length distribution of 370 ± 60 nm. This was followed by a 5 min ramp-up period to the optimal growth temperature for GaP growth (560 °C), during which TEGa and *tert*-butylphosphine (TBP) line pressures were adjusted to the pressures needed for GaP growth. Next, the GaP segment was grown for 20 min, which resulted in a 900 ± 110 nm long GaP segment. Following this, two 30-s-long growth interruptions (GIs) were employed to stabilize the TBAs and TBP line pressures for GaAs_*x*_P_1–*x*_ QD growth, as described in the alloy calibration section. The need of employing two growth interruptions comes from the fact that a single growth interruption, with neither a linear nor a steplike change in precursor line pressures, is enough to have stable line pressures when starting the QD growth. Additionally, a single but longer GI could destabilize the nanoparticle (NP). After the QD growth, a GaP tip was finally grown using the same line pressures and temperature as the previous GaP segment, resulting in a total NW length in the 1.5–2.5 ± 0.2 μm range, depending on the tip growth duration of the different samples. In the case of NWs with a shell, radial GaP growth was achieved around these QD NWs by stepwise reduction of the growth temperature by 30 °C, coupled with the ramping up of precursor line pressures for enhanced radial growth, as explained later.

In the following, we show the GaP tip morphology optimization, the GaAsP alloy composition calibration, and finally the realization of the optimal QD NW samples, with and without a GaP shell.

### 2.1 Morphology Optimization of the GaAs/GaP NW-Heterostructured Stems

This investigation aimed to optimize the morphology of the GaAs/GaP-heterostructured stems for subsequent QD insertion. All samples were grown using the Au-film dewetting procedure to create Au NPs (see the Experimental Methods section for more details).

**Figure 2:**
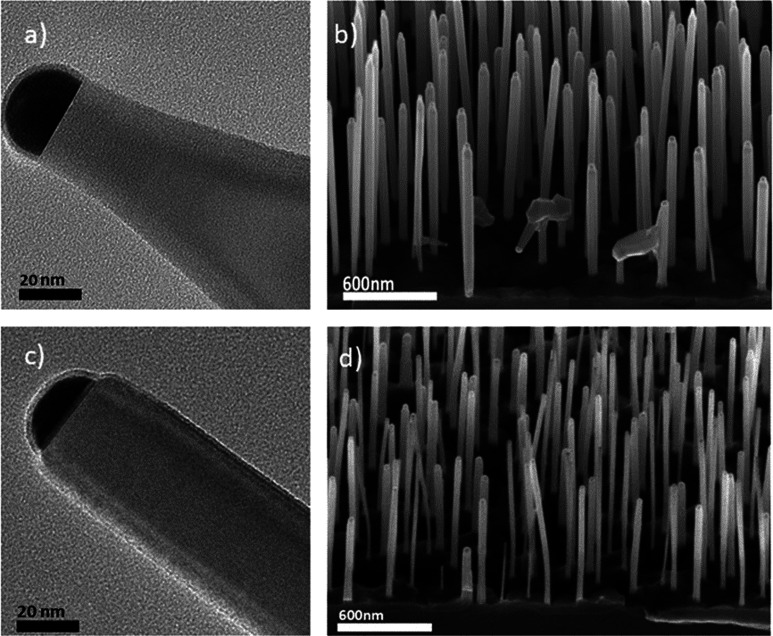
GaAs/GaP NW-heterostructured stems obtained from Au-film dewetting, using different growth conditions. (a) Bright-field TEM image of a single GaP tip obtained using 0.7 and 2.5 Torr of the TEGa and TBP line pressures, respectively. (b) 45°-tilted SEM image of the ensemble of NWs obtained with the same line pressures as those in part a. (c) Bright-field TEM image of a GaP tip obtained using the reduced pressures (0.4 and 2.0 Torr of TEGa and TBP line pressures). (d) 45°-tilted SEM image of the ensemble of NWs obtained using the same line pressures as those in part c and shortening the GaAs and GaP growth times. The TEM images refer to single NWs transferred from the NW ensembles shown in the SEM images.

Based on previous studies,^21^ it is well-established that GaP NWs grown at high TBP pressure exhibit a pure WZ crystal structure. We determined that maintaining the TEGa and TBP line pressures at 0.7 and 2.5 Torr, respectively, results in NWs with nearly perfect crystal purity. However, these NWs exhibit a pencil-shaped tip, as shown in Figure 2a,b. This morphology may influence the subsequent QD growth, as is explained in Section S1. The observed pencil-shaped morphology is likely attributed to vapor–solid growth on the NW sidewalls and to the short Ga adatom diffusion length.^23^ This morphology may lead to the formation of a cone-like GaAsP quantum well beneath the QD (Section S1 and Figure S1), thereby compromising its carrier confinement. To address this issue and obtain untapered NW tips, we employed three approaches. (i) Temperature optimization: increasing the temperature during GaP segment growth to increase the Ga adatom diffusion length. (ii) Precursor pressure reduction: lowering precursor line pressures to decrease the probability of Ga adatom nucleation on the NW sidewalls. (iii) NW length control: shortening the lengths of the GaAs stem and GaP segment in order to grow the QD at a height comparable to the diffusion length of Ga adatoms.

While the temperature increase did not significantly alter the tip morphology within the available temperature range to maintain the GaP axial growth (about 60 °C), reducing the precursor pressures led to a substantial improvement: GaP NWs grown using TEGa and TBP line pressures of 0.4 and 2.0 Torr, respectively, exhibited approximately 60% of the NWs with untapered tip morphology, as is visible in Figure 2c.

Literature suggests^15^ that radial growth is a stochastic-driven process, connected to the ratio between the diffusion length of Ga along the NW sidewalls and the NW length. By limiting the total NW length beneath the QD to less than 1 μm, which is roughly the diffusion length of Ga,^24,25^ we managed to prevent radial growth of GaAsP on the NW sidewalls. Indeed, by reducing the GaAs/GaP stem length from 1200 ± 280 to 940 ± 130 nm, we obtained a significant improvement, reaching a yield of NWs with untapered tips approaching 100%, as is visible in Figure 2d.

These NWs were analyzed by transmission electron microscopy (TEM) and electron diffraction, showing a WZ crystal structure almost free of stacking faults. The GaP unit cell was determined by three-dimensional electron diffraction (3DED).^26^ Data were collected in scanning transmission electron microscopy (STEM) mode using the higher spot size and a condenser aperture of 10 μm. The CL3 lens current was modified to have a quasi-parallel beam on the sample, with a diameter of about 30 nm. 3DED data were collected in a tilt range of ±30°, with a tilt step of 1° and an exposure time of 0.5 s. The camera length was 250 nm. Data were analyzed by *PETS2* software.^27^ The GaP cell was determined as a hexagonal primitive (hP), with *a* = 3.89 Å and *c* = 6.40 Å; the *c* axis is parallel to the NW growth direction.

In summary, through controlled growth conditions (a GaP growth temperature of 560 °C, line pressures of 0.4 and 2.0 Torr for TEGa and TBP, respectively, and a total stem length of 940 ± 130 nm), we have successfully optimized the morphology of the GaAs/GaP NW-heterostructured stems for QD growth, achieving the desired shape and a high crystal quality.

### 2.2 Group V Incorporation in GaAs_*x*_P_1–*x*_ Ternary Alloy

The tunability of the QD emission wavelength through manipulation of the GaAs_*x*_P_1–*x*_ ternary alloy composition is a core aspect of our study. To investigate this, we explored a wide range of metal–organic precursor line pressure ratios to achieve precise control and tunability of the QD composition, i.e., *x* from 0.1 to 0.9.

**Figure 3:**
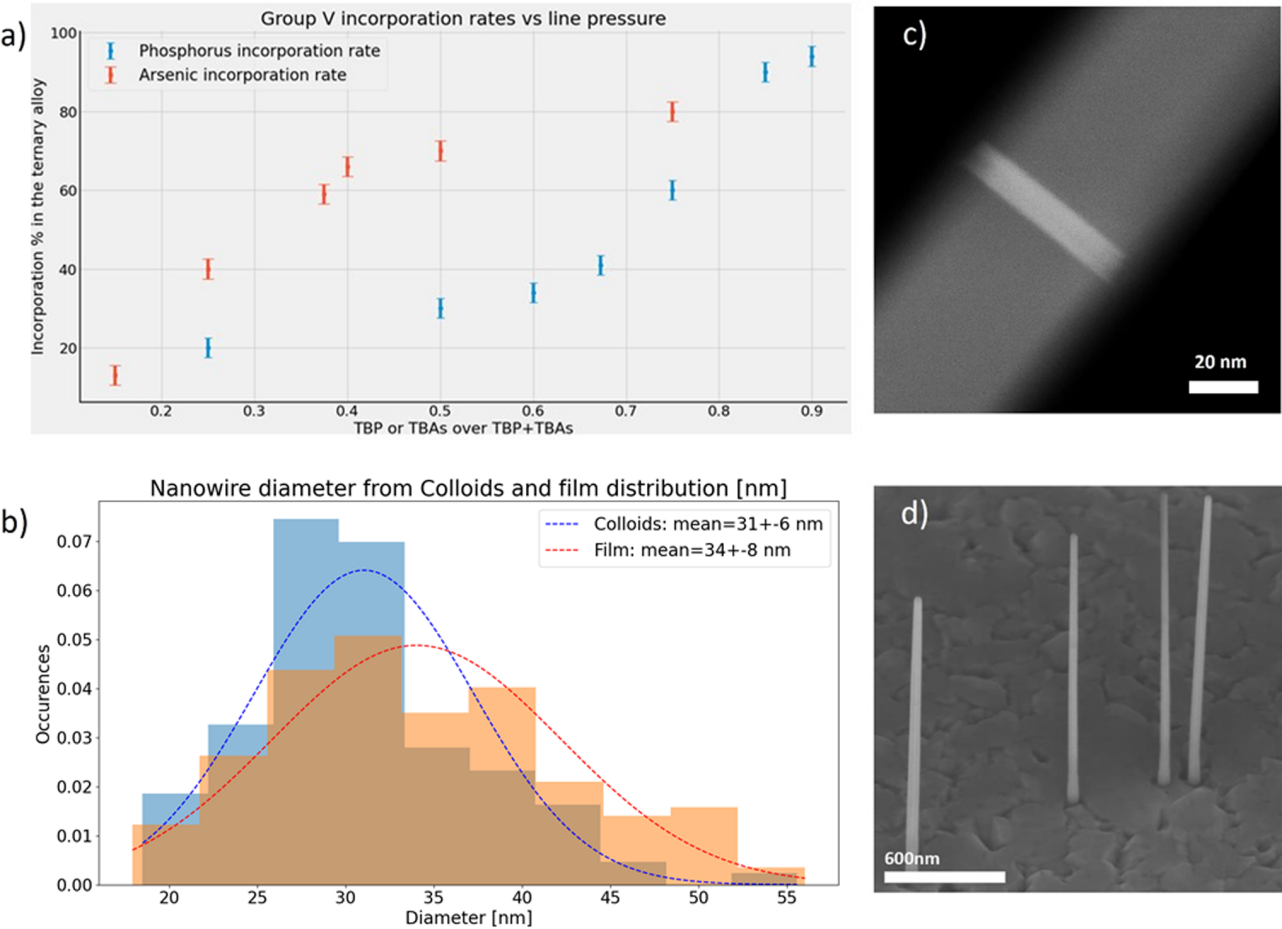
(a) Group V percentage in the ternary alloy as a function of the partial Group V precursor line pressure measured on NWs grown from Au-film dewetting. (b) Diameter distribution of the NWs grown from colloids vs those grown from Au-film dewetting. (c) TEM image of a GaAs_*x*_P_1–*x*_ QD with *x* = 0.90 embedded in a NW grown from the Au 20 nm colloidal solution, where the sharp interfaces are to be noted. (d) 45°-tilted SEM image of the ensemble of NWs grown from a colloidal solution.

Due to the limited spatial resolution of the energy-dispersive X-ray analysis (EDX) at SEM, which renders the accurate measurement of the QD composition impossible, we grew several hundred-nanometers-long segments of GaAs_*x*_P_1–*x*_ on top of the GaAs/GaP NW-heterostructured stems, keeping the TEGa line pressure at 0.4 Torr and using different TBAs and TBP line pressures, and we measured their compositions. For the sake of consistency, we maintained a constant III/V ratio for all of the samples by keeping the sum of the two Group V line pressures fixed at 2 Torr. The results of the incorporated As and P % for the NWs grown from Au-film dewetting are illustrated in Figure 3a. It is observable that the two species have different incorporation rates in the ternary alloy as a function of their correspondent precursor partial pressure: TBAs/(TBP + TBAs) for As incorporation (red squares) and TBP/(TBP + TBAs) for P incorporation (blue circles). Similar calibration procedures have been extensively conducted in both 2D systems^28^ and quasi-1D NW systems,^29^ confirming the reliability of our approach.

For each as-grown sample, we measured several NWs, and the composition was uniform with a standard deviation of 2–3 atom %. However, our SEM–EDX system has an error of about 5% on the quantification of the alloy composition. We have therefore used 5% as the error of the measurement; that is why higher percentage compositions have bigger error bars.

The measured composition values are then transformed into the stoichiometric composition of the ternary alloy, and the error is propagated accordingly.

Our findings, aligned with other reports, indicate that the incorporation rates of P and As in the ternary GaAs_*x*_P_1–*x*_ alloy are different and that the percent of As incorporated in the alloy does not scale linearly with the TBAs line pressure.

We have therefore demonstrated very good control and full-range tunability of the composition of the GaAs_*x*_P_1–*x*_ ternary alloy. For the QDs growth, three compositions were selected, all with high As % (in order to maximize the band-gap energy difference with the GaP barriers), namely, *x* = 0.5, 0.7, and 0.9, and their emission properties were investigated by μ-PL, as discussed in the last section.

### 2.3 Growth from Au Colloids and Radial Growth Dynamics

It is known that surface states can hinder the optical and electrical properties of QDs and quantum wells; in particular, they can quench the intensity of the emitted radiation.^30^ For this reason, we decided to grow a GaP shell around the GaAs/GaP/GaAsP/GaP QD NWs. For this step, we chose to use the NWs grown from a diluted solution of 20 nm Au colloids instead of Au-film dewetting to decrease the NW density and avoid any shadowing effect between neighboring NWs, which is known to strongly decrease the radial growth.^31^

Importantly, the NWs obtained from Au colloids have a narrower diameter distribution compared with the NWs obtained from Au-film dewetting,^32^ as is visible in the diameter distribution displayed in Figure 3b. Because the axial growth rate is related to the NP diameter,^33^ narrower NP diameter distributions lead to narrower NW length distributions. As a consequence, with Au colloids, we achieved a more uniform NW length and QD thickness compared to Au-film dewetting.

Parts c and d of Figure 3 show images of NWs grown from colloidal solution deposition without the GaP shell. Panel c shows a STEM image of a portion of a single QD NW, and panel d shows a 45°-tilted SEM image of the as-grown GaAs/GaP/GaAsP/GaP NW. A notable feature is the presence of sharp interfaces both between the lower GaP stem and the QD and between the QD and the GaP segment above it along the growth axis. The NW density is 2 orders of magnitude lower with respect to the ones obtained from the Au-film dewetting procedure (5 × 10^–1^ vs 5 × 10^1^ NW/μm^2^) while maintaining the proper untapered morphology at the same precursor pressures and temperature.

These QD NWs have an average length of 1500 ± 120 nm and an average diameter of 40 ± 8 nm at the position of the QD. With STEM imaging and STEM–EDX analysis (see more about this in Section S2), we can say that the interfaces between the GaP segments and the GaAsP QD are sharp within the instrumental resolution (Figure S2) and the NWs are mostly untapered (more on this in the paragraph below). Being that WZ GaP NWs grown along the (111)B direction have a hexagonal cross section with six equivalent {110}^21^ sidewalls that in our case are perpendicular to the hexagonal faces, and the NWs are untapered, the QDs are almost perfect prisms, which is a good morphology for optical properties.

Defining the tapering ratio of a NW as the ratio between the diameter right underneath the Au particle and the diameter at the NW base, a perfect cylinder will have a ratio of 1. In our case, this ratio after the morphology optimization of the NWs grown with the Au film shown in Figure 2c is 1.21 (while for the pencil-shaped NWs in Figure 2a, it is 2.48), leading us to conclude that the optimized NWs are almost untapered. However, the tapering ratio of the NWs obtained with the Au colloids is 1.43, meaning that a thin radial deposition occurs during the GaP tip growth using low-density Au colloids, and this results in a few-nanometer-thick passivation shell around the QDs. This is a nonintentionally grown GaP shell, but it could already be good for the QD emission. To intentionally grow a thicker shell, different growth conditions must be employed. We found that lower growth temperatures and higher line pressures promote radial growth at the expense of axial growth. A preliminary investigation of the radial growth rate dynamics is reported in Section S3, where we show the parameters that affect the radial growth and the final NW morphologies (Figures S3–S5). This poses the base for a future careful optimization of the radial growth in order to obtain the NW antenna geometry to have a waveguide effect of the QD emission.^12^

### 2.4 Optical Emission Properties

In this section, we will discuss the optical properties of the optimized QD NWs grown by Au colloids, investigated through μ-PL measurements.

#### 2.4.1 Energy Emission Tunability

Here we show the emission properties of GaAs_*x*_P_1–*x*_ QDs with similar thickness (12 ± 6 nm) and two different compositions: 70% and 90% As content, on single NWs and NW ensembles.

**Figure 4:**
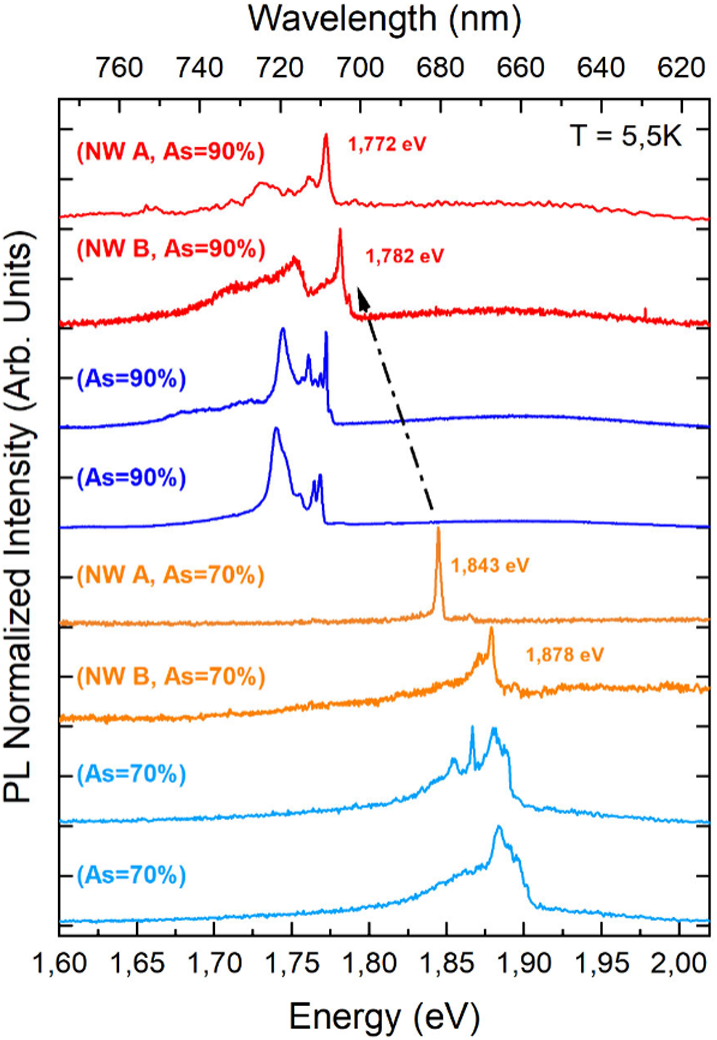
μ-PL spectra on ensemble (cold colors, two representative spectra for each sample, acquired on different points) and single (warm colors) GaAsP QD NWs at *T* = 5.5 K. Single NWs were manipulated and measured on silicon, with the axis parallel to the substrate, and NW ensembles were measured in the vertical position (see the Experimental Methods section). NW A and NW B show representative spectra of two single NWs detached from the same sample, i.e., with the same As content. The emission energy redshifts as a function of the As %, as indicated by the dotted black arrow.

In Figure 4, we present representative μ-PL spectra of two single NWs (warm colors) and of two positions of the NW ensembles (cold colors) measured at 5.5 K. The QDs have similar thicknesses and different compositions, as displayed in the legend. The ensemble spectra show an emission band that characterizes the sample as a function of the incorporated As content: a clear shift is visible from 1.87 eV (As = 70%) to 1.76 eV (As = 90%), in qualitative agreement with our preliminary calculations performed in the ZB phase, resulting in 1.89 eV (As = 70%) and 1.76 eV (As = 90%). Calculations in the WZ phase were not possible because carriers’ effective masses and refractive indices for the two compounds in the WZ phase are either not experimentally available or do not agree with the theory. Despite the approximation arising from considering the ZB phase, these calculations are still useful for understanding the magnitude and direction of the energy shift as a function of As % and the QD size. Emission from the NW ensembles is a broad-band convolution of numerous narrower lines arising from the multiple NWs illuminated in this configuration. We performed preliminary calculations to estimate the contributions to the broad fwhm of the ensemble signal. We estimated the variation in the emission energy due to variations in the QD size. Considering that the QD thickness, as measured by STEM, is 9 ± 3 nm for 90% As content and 15 ± 3 nm for 70% As content, our calculations return variations in the QD emission energy respectively of ±25 and ±10 meV. Fluctuations in the As content in the QD can also contribute to shifting the emission from wire to wire. EDX measurements show invariance, within the experimental error of 5%, between the As content in the QDs of the same sample. Considering fluctuations in the As content by ±2.5%, we calculated a variation in the emission energy of ±15 meV for 90% As content and ±10 meV for 70% As content. Therefore, in the ensemble spectra in Figure 4, fluctuations in the QD size and As content are both likely to contribute to the total fwhm. Last, because smaller QD size results in higher quantum confinement, decreasing the size of the 15 nm QD (70% As content) would further increase the emission tunability range.

The μ-PL spectra of individual NWs are characterized by a single narrow peak, whose energy decreases with increasing As %, as discussed for the ensemble measurements. The spectral position and fwhm of the QD peak of all single NWs in the same sample slightly vary, as discussed, and the QD peak energy resides well within the ensemble spectral emission range. Measurements on a large number of individual NWs allowed us to estimate more precisely the average emission energy through the “Lorentzian” fit of the main emission peaks: 1.767 ± 0.013 eV for As = 90% and 1.858 ± 0.029 eV for As = 70%. These values are in agreement with previous measurements on GaAsP QDs in ZB GaP NWs,^20^ but in our samples, we have precisely estimated the As % incorporated in the QD, so we could correlate the QD composition with the emission energy.

In addition to the data presented here, we performed μ-PL measurements on single QD NWs with a 30-nm-thick GaP shell (as shown in Section S4). The comparison between NWs with a thick GaP shell and NWs with only a thin passivation layer (as explained in section 2.3) shows that there is no clear improvement in the QD emission intensity: the shell results in the appearance of a broad emission band underlying the QD peak (Figure S6). This is likely due to conventional impurities/defects incorporations formed in the shell during the low-temperature GaP growth. The capability to control the shell quality and thickness will be crucial in view of a future optimization to obtain the waveguide effect. At this stage, the NWs with only the passivation layer show better optical quality, exhibiting a narrower and more spectrally isolated QD emission peak; therefore, all of the measurements in the following sections are performed on a NW without a GaP shell.

We also performed μ-PL measurements on pure GaP NWs with no QDs. These measurements allowed us to study the emission properties of WZ GaP, including the phonon replicas, and also provided a clear picture of the low-temperature spectrum of the material (Section S5). The emission spectra of GaP NWs (Figure S7) show a broad band at 1.89 eV that we attribute to defect and impurity states. Furthermore, we observed numerous narrow intense lines in the range 2.05–2.2 eV. Power-dependent studies allowed us to recognize these peaks as single and charged excitonic radiative recombinations due to impurity atoms incorporated during growth. We were able to identify the phonon replicas of these excitonic peaks (Section S5, Table S1, and Figure S8), involving the TA (Δ*E* = 14 meV) acoustic phonon mode and the TO (Δ*E* = 45 meV) and LO (Δ*E* = 50 meV) optical phonon modes.^34^ Temperature-dependent measurements (Figure S9) showed fast quenching of the intensity of the excitonic peaks, with an estimated activation energy between 3 and 6 meV.

Finally, we studied the emission spectrum of a QD sample with 50% As content (Section S6). This sample does not show a clear QD-like narrow emission line (Figure S10) probably due to the weak confining potential for this low As content (arising from the high GaAs_*x*_P_1–*x*_ band-gap energy compared to NWs with 70% and 90% As content): photogenerated carriers do not get trapped in the shallow potential of the GaAs_*x*_P_1–*x*_ QD and mostly recombine in the GaP barriers. In samples with QDs with 70% and 90% As, a deep confining potential is instead established; thus, carriers radiatively recombine in the QD region.

#### 2.4.2 Spatial Localization of the Light Emitter

**Figure 5:**
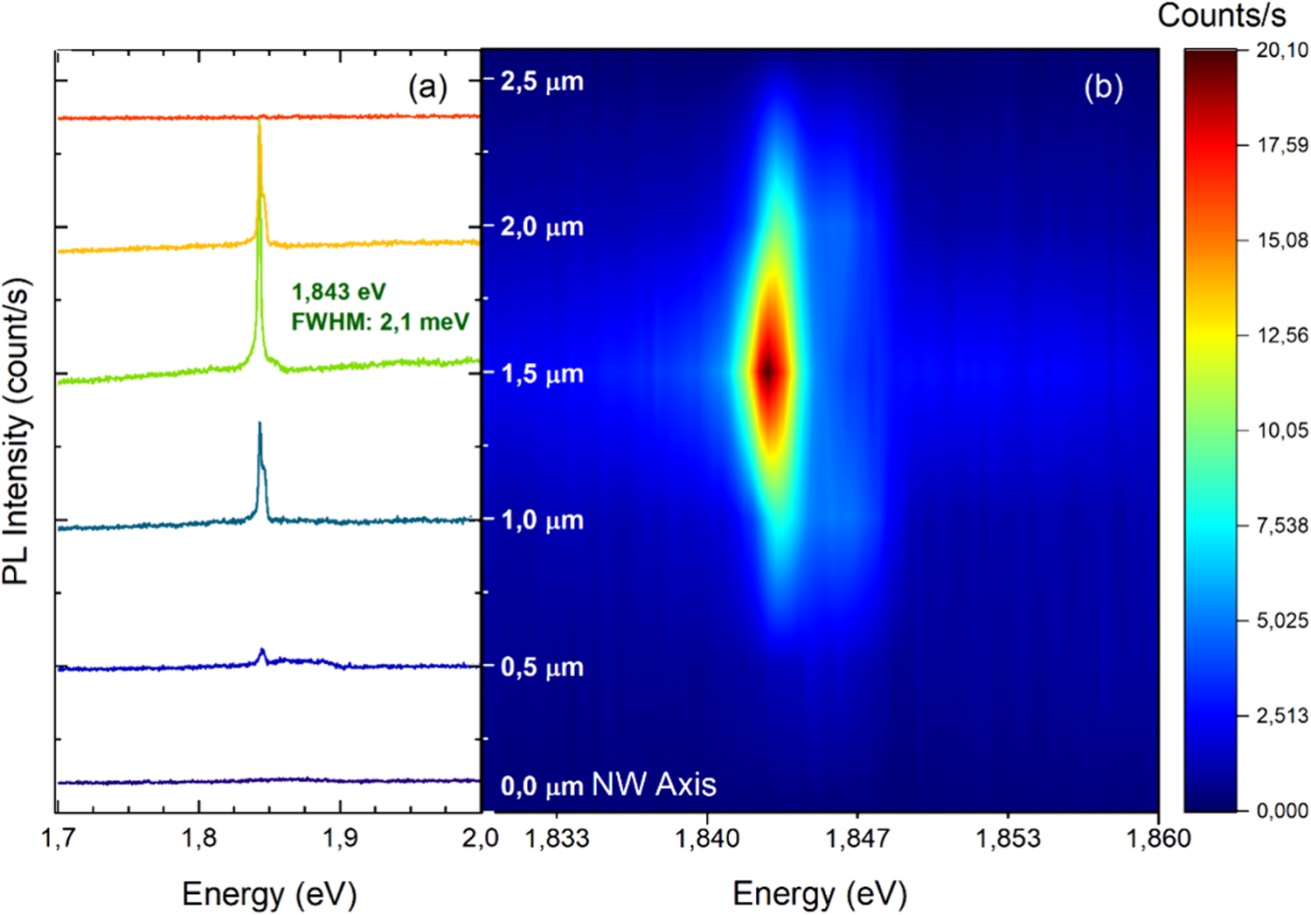
μ-PL measurements of a single GaAsP QD NW with As = 70%, *T* = 5.5 K, and an excitation power of 1 μW. (a) μ-PL spectra along the NW axis. The QD signal is visible as an intense and sharp emission line at 1.843 eV with a fwhm 2.1 meV. (b) Color plot of the intensity of the emission along the NW axis, with a close-up of the QD emission spectral range. Single spectra singled-out by this plot are shown in panel a on a wider spectral range. Measurements show the locality of the emitter, whose intensity is localized in a region smaller than 1 μm.

To study the variation in emission along the NW axis and to confirm that the intense and narrow line observed in the individual wires is attributable to the QD emission, we carried out μ-PL maps on individual NWs. Parts a and b of Figure 5 show a representative map and relative spectra taken at 5.5 K with a constant excitation power of 1 μW. The NW is ∼2.3 μm long and is singled out from the ensemble of QD NWs with 70% As content. Panel a presents the spectra acquired by moving with steps of 500 nm from one end of the wire to the other. We observe an intense emission line at 1.843 eV (in agreement with spectra shown in Figure 4) with a fwhm of 2.1 ± 0.2 meV. Mechanisms that may contribute to the broadening of our QD emission peak are point and line defects, interface defects, and alloy disorder. Our linewidth is comparable to similar QDs in this material system, as summarized in the Introduction. Moving away from the narrow QD peak, we observe an underlying broadband emission at energies between 1.84 and 1.89 eV, probably originating from impurity states in the GaP passivation shell, such as C and Sb (see spectra on GaP NWs in Sections S5 and S6).^35^

The color plot in panel b shows the spatial distribution along the NW axis of the main peak (1.843 eV). It gains intensity in a localized area of comparable size, with the width of the spot illuminating the wire, confirming the presence of a smaller structure that we have identified as the GaAsP QD. As mentioned in section 2.1, the QD is located in the approximate center of the NW growth axis, as is also visible in the μ-PL map. Hence, parts a and b of Figure 5 demonstrate again the creation of an efficient and localized carrier capture mechanism. A similar result is obtained also for the As = 90% QDs, as shown in Section S7 and Figure S11).

#### 2.4.3 Power and Temperature Study

**Figure 6:**
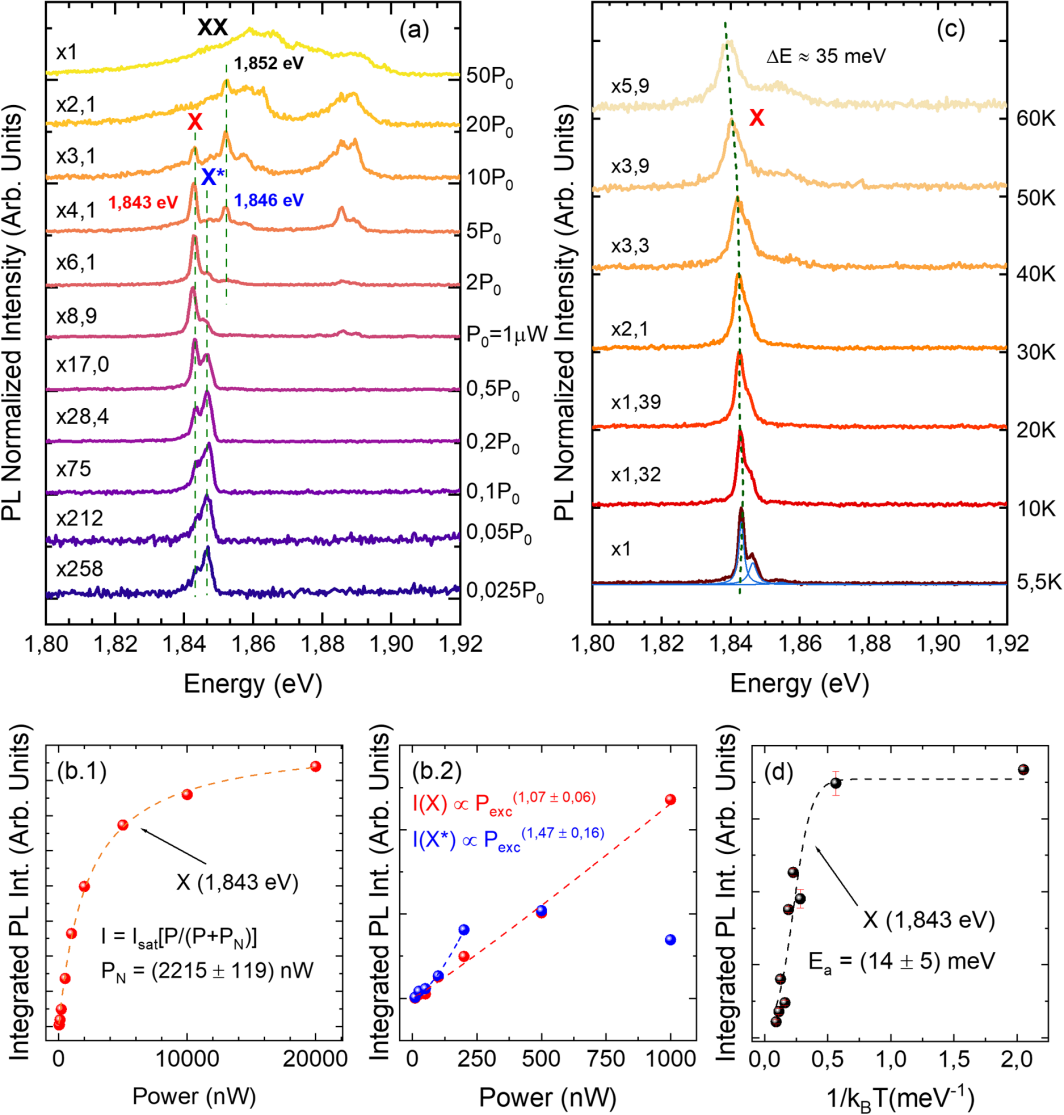
Power- and temperature-dependent μ-PL measurements on single GaAsP QD NWs with As = 70%. (a) Power-dependent spectra of the QD peak acquired at 5.5 K, with intensity normalization factors indicated on each spectrum. Dashed lines highlight the single exciton (X), charged exciton (X*), and biexciton (XX) emission peaks. *P*_0_ = 1 μW. (b.1) Plot of the integrated intensity (obtained from the spectra in panel a of peak X as a function of the power. The dashed line shows a fit with , where *P*_N_ is the laser power at which the intensity is half of *I*_sat_. (b.2) Power-dependent integrated intensity of X (red dots) and X* (blue dots) at powers below *P*_0_ to show the X* saturation curve and X linear behavior. (c) Temperature-dependent μ-PL spectra, acquired at *P*_0_, with normalization factors. The blue curves under the 5.5 K spectrum show the Lorentzian curves used to fit each peak. The green dashed line highlights the thermal red shift of the emission. (d) Integrated PL intensity of the X peak as a function of the reciprocal of the temperature with the Arrhenius fit (dashed line) and extracted activation energy.

In this section, we discuss the evolution of the emission of single GaAsP QDs having 70% As content as a function of the excitation power and temperature. Although challenging, this allowed us to study the nature of the emitters and the distribution of the energy states. Figure 6a shows μ-PL spectra as a function of the laser power, with given normalization factors, highlighting the gain in intensity as the excitation power increases. Measurements were carried out on the same QD presented in Figure 5. Below the power of *P*_0_ = 1 μW, the spectra are dominated by a narrow band composed of two main peaks: for excitation powers lower than 0.2*P*_0_, we observe the predominance of the high-energy feature at 1.846 eV, which then saturates at 0.5*P*_0_ and exchanges intensity with the low-energy peak at 1.843 eV. The latter becomes dominant at *P*_0_, and at this power, we find indeed the characteristic spectrum presented in the map. We fitted the integrated intensity of both peaks with the power law: *I*_exc_ ∝ (*P*_exc_)^M^ (eq 1), as plotted in Figure 6b.2 for powers below *P*_0_. For the lowest energy peak, we find a slope of *M*_X_ = 1.07 ± 0.06, which indicates a single excitonic recombination process (thus, we label that peak as a free exciton, X). This assumption is further confirmed by the invariance of the emission energy as a function of the excitation power (see a dashed line labeled X in Figure 6a) and by the saturation of emission at high excitation power presented and fitted in Figure 6b.1. For the higher energy state (1.846 eV), we estimated a slope of the power law of 1.47 ± 0.16, suggesting the presence of a charged exciton (X*, positive or negative) resulting from the unequal capture of photoexcited carriers in the QD.^36^

As shown in Figure 6a, at higher excitation powers, we also see the population of high-energy states.^36^ At 2*P*_0_, a peak of 1.852 eV starts to gain intensity without redshifting. The integrated intensity fit for this contribution yields a power law coefficient of 2.3 ± 0.1; therefore, we attribute this peak to a biexcitonic recombination process (XX; see the dashed line). We can also observe a double peak centered at 1.887 eV arising at *P*_0_ and gaining in intensity with increasing power. This peak is visible in most of the single NWs measured and could originate from radiative recombination from excitons in the p-shell states of the QD.^37^ The integrated intensity trend fitted with the power law yields 1.01 ± 0.05 eV for the peak at 1.886 eV and 1.15 ± 0.05 eV for the peak at 1.889 eV, respectively. At 50*P*_0_, multiexciton processes start to gain in intensity and do not allow us to spectrally resolve single peaks.^36^

Figure 6c shows temperature-dependent measurements on the same QD NW, with multiplication factors showing the intensity loss as the temperature rises. We observe that the X peak gets broader and the luminescence intensity quenches at higher temperatures, as expected for a single free exciton. The energy states related to higher energy emission start to populate with photogenerated carriers because they gain thermal energy with an increase in the temperature, and at around 40 K, it is no longer possible to spectrally resolve X from the other contributions to the emission spectrum (such as the X* peak). As a result, the emission broadens (a fwhm of 7.4 meV at 60 K) and undergoes an expected thermal redshift with a Varshni-like behavior^38,39^ of 35 meV from 5.5 to 60 K. Similar studies for the As = 90% QD NWs are displayed in Section S7 and Figure S12, even in a larger temperature range, up to 200 K.

The “Arrhenius” plot of the integrated PL intensity of the X peak in Figure 6c is shown in part d. The data are fitted considering one active nonradiative recombination channel for the exciton: we estimate the activation energy *E*_a_ = 14 ± 5 meV, suggesting that, due to the thermal energy, carriers escape to an excited state located ∼14 meV beyond the free exciton, most likely the biexciton, also visible in the power study at high powers and in the temperature study at temperatures above 40 K.

## 3 Conclusions

In this study, we investigated the growth and optical properties of WZ GaP NWs with single GaAs_*x*_P_1–*x*_ QDs. We have shown a great degree of control over the NW morphology and the QD chemical composition. Moreover, we have found the growth parameters that affect axial and radial growth. Single-NW μ-PL measurements show how the emission is dominated by a narrow peak whose emission shifts according to the As content of the QD, which makes the system a promising tunable quantum light source. μ-PL mapping along the NW axis confirms the attribution of this peak to the QD and highlights the realization of a localized and efficient carrier recombination mechanism that promotes emission from the QD rather than from the GaP NWs. Power-dependent measurements revealed the presence of two radiative mechanisms contributing to the QD emission. In the low-excitation-power regime, the spectrum is dominated by the emission of a charged exciton. This feature loses intensity as we raise the excitation power, while the single free exciton gains in intensity. Finally, the study of the QD emission as a function of the temperature shows quenching and a Varshni-like shift of the main peak. However, the peak bandwidth is still <8 meV at 60 K.

Overall, our findings demonstrate that high-quality light emitters can be realized through optimization and control of the growth process and underline the potential for these novel WZ GaAs_*x*_P_1–*x*_ QD NWs with tailored composition to advance applications in quantum optics and photonics in the future.

## 4 Experimental Methods

The QD NWs were synthesized by chemical beam epitaxy utilizing a Riber Compact-21 system on GaAs(111)B substrates, employing Au-assisted vapor–liquid–solid growth.^33^ Triethylgallium (TEGa), *tert*-butylarsine (TBAs), and *tert*-butylphosphine (TBP) were used as gaseous metal–organic precursors, and it is possible to set the line pressures as a reference for the fluxes introduced in the chamber. The substrate temperature was monitored via a pyrometer and cross-validated with a manipulator thermocouple, ensuring an overall accuracy of ±10 °C. Catalyst NPs were fabricated by employing two distinct methods: 0.02 nm thin Au-film dewetting and deposition of 20 nm Au colloids (BBInternational EM.GCnn) via drop-casting onto bare substrates.

Thin Au-film deposition on the substrates was conducted within a high-vacuum (10^–5^ Torr) thermal evaporation chamber and monitored via a thickness monitor. After deposition, the dewetting process was initiated within the growth chamber at 500 °C under 1 Torr of TBAs line pressure.

For Au colloidal solution deposition, the substrates underwent a preliminary treatment involving immersion in a 0.1% poly(l-lysine) solution for 30 s, followed by rinsing in deionized (DI) water and nitrogen drying. The colloidal solution was then drop-cast onto the substrate surface, followed by a DI water rinse and nitrogen drying.

Postgrowth characterization involved imaging via scanning electron microscopy (SEM; Zeiss Merlin operated at 5 kV), employing both top-view and 45° side-view orientations of the as-grown samples, to comprehensively assess the morphology and dimensions of the NWs. The GaAsP alloy composition was measured through energy-dispersive X-ray analysis (EDX) employing a Bruker Quantax EDS system mounted on a Zeiss Ultraplus scanning electron microscope. Transmission electron microscopy (TEM), scanning transmission electron microscopy (STEM), STEM–EDX, and three-dimensional electron diffraction (3DED) were conducted using a JEOL JEM-F200 Multipurpose microscope, working at 200 kV and equipped with a Schottky field-emission gun and a silicon-drift detector. TEM images were recorded with a GATAN RIO16 CMOS camera, and 3DED data were recorded with an ASI CHEETAH hybrid-pixel detector.

Geometric parameters of the NWs were measured using the image analysis software *ImageJ*, while data analysis was executed utilizing a custom-written code interfaced with Python libraries.

Low-temperature μ-PL measurements were performed by utilizing a closed-cycle helium cryostat operating at 5.5 K, in which the samples were placed on a piezoelectric stage. As an excitation source, a 532 nm solid-state laser was used with controlled excitation power to avoid damage to the NWs. The light was focused through a 100× objective with a numerical aperture of 0.75, resulting in a diffraction-limited spot size of 750 nm.

The signal was collected in a backscattering configuration and dispersed by a spectrometer with a focal length of 50 cm with a grating groove density of 300 grooves/mm and directed to a liquid-nitrogen-cooled silicon CCD detector. We measured both NW ensembles and single NWs that we took individually with micromanipulators and positioned flat on a patterned silicon substrate. During measurement of single NWs, the μ-PL signal wavevector is perpendicular to the NW long axis, as opposed to being parallel to the NW long axis during measurement of ensembles of standing wires on the growth substrate.

## Supporting Information Available

Section 1 on pencil-shaped tips, Section 2 on interface abruptness, Section 3 on GaP radial growth, Section 4 on shell effect, Section 5 on GaP emission, Section 6 on GaAsP QD NWs – As = 50%, Section 7 on additional optical spectroscopy data, and Section 8 on calculation of the exciton bohr radius (PDF)
