# Supplementary material for: Tunable GaAsxP1–x Quantum-Dot Emission in Wurtzite GaP Nanowires
Source: ACS Appl Mater Interfaces. 2024 Nov 13;16(47):65222–32. doi: 10.1021/acsami.4c15343 (PMC11615845; doi:10.1021/acsami.4c15343)
Supplement: Supplementary file 1 — am4c15343_si_001.pdf [file am4c15343_si_001.pdf]

Supporting Information

Tunable $GaAs_xP_{1-x}$ quantum dot emission in wurtzite GaP Nanowires

Robert Andrei Sorodoc[1#], Paolo De Vincenzi[2#], Akant Sagar Sharma[2], Giada Bucci[1], Mario Roggi[2], Enrico Mugnaioli[3], Lucia Sorba[1], Marta De Luca[2] and Valentina Zannier[1]*

[1] NEST Istituto Nanoscienze-CNR and Scuola Normale Superiore, Piazza S. Silvestro 12, 56127 Pisa, Italy.

[2] Department of Physics, Sapienza University of Rome, P.le A. Moro 5, 00185 Rome, Italy

[3] Department of Earth Sciences, University of Pisa, Via S. Maria 53, 56126 Pisa, Italy

[#] These authors contributed equally to this work.

** email: valentina.zannier@nano.cnr.it*

Contents

S1. Pencil-Shaped Tips

S2. Interface Abruptness

S3. GaP Radial Growth

S4. Shell Effect

S5. GaP Emission

S6. GaAsP QD NWs – As = 50%

S7. Additional Optical Spectroscopy Data

S8. Calculation of the Bohr Exciton Radius

S1. Pencil-Shaped Tips

In this study, we present a crucial aspect of our research that underscores the necessity of optimizing the morphology of the GaP tip before the GaAsP quantum dot (QD) insertion.

Figure S1 shows a a dark-field STEM image (panel a) with the energy-dispersive X-ray (STEM-EDX) analysis and a bright-field TEM image (panel b) of the GaAsP QD obtained on top of the GaP stem with a pencil-shaped tip grown using 0.7 and 2.5 Torr of TEGa and TBP line pressures. Panel c) shows a schematic representation of the NW heterostructure obtained at these growth conditions.

An elongated "tail" associated with the QD is visible from the image contrast (panel a). Moreover, in the EDX line profile of this segment, the Arsenic (blue line) intensity along the NW axis clearly shows that the shell around the tip of the first GaP segment is made of GaAsP. This suggests the potential for carrier recombination within these layers, instead of the axial QD.

As explained in the main text, and from known literature, tapered tips in NWs are associated with the radial growth typically occurring in Ga-V systems, initiated by the stochastic nucleation on the NW sidewalls. Therefore, we could expect to have GaAsP radial growth simultaneously occurring with the axial QD growth if we have radial growth during the GaP segment growth. Figure S1 indeed confirms this hypothesis.

Given the critical importance of spatial confinement for QDs acting as single photon sources, it becomes imperative to pursue an untapered NW tip morphology. From this observation comes the need for the morphological improvement that we achieved, as described in the main text.

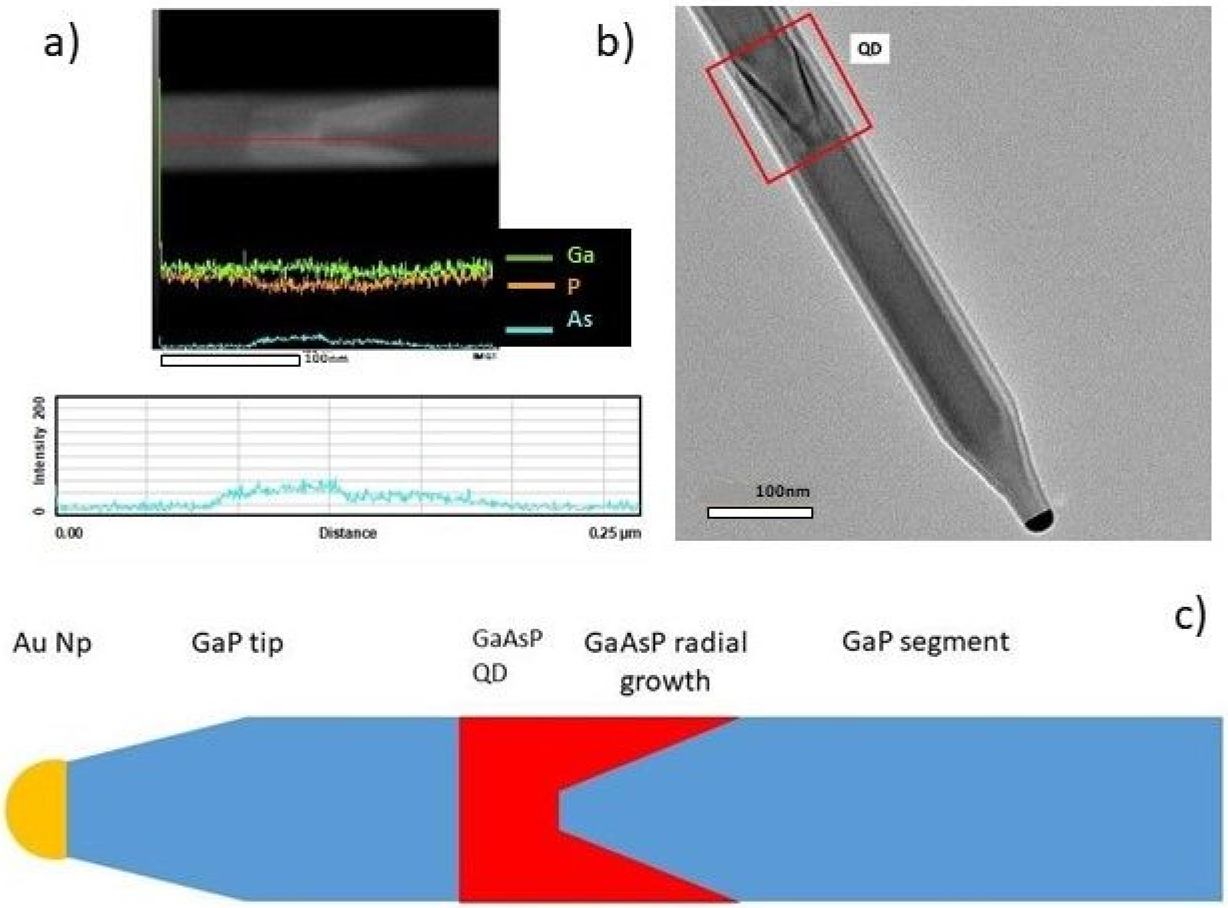

Figure S1: a) STEM-EDX image of a GaAsP QD with 70% As within a GaP NW (GaP segment grown with 0.7/2.5 TEGa/TBP line pressure ratio). The green line is the Ga intensity, the orange one the P intensity while the blu one is the As intensity. The GaAsP tail radially grown on the pencil-shaped tip of the GaP segment is visible. b) Bright-field TEM image of a GaAsP QD and the tip of the NW heterostructure. Both a and b images were taken from the same sample. c) Schematic representation of the structure with blue color representing GaP and red color representing GaAsP. Not to scale.

S2. Interface Abruptness

Figure S2 displays an EDX line profile of the As signal of a GaP/$GaAs_{0.9}P_{0.1}$/GaP sequence in a NW grown with the optimized TEGa/TBP line pressure ratio for GaP, namely 0.4/2.0, resulting in propert tip morphology, as discussed in the main text. The intensity of the As signal drops abruptly down to zero at both sides, indicating sharp interfaces between the GaAsP QD and the upper and lower GaP segments. The signal is quite noisy because we had to be very fast in the acquisition time, to avoid any drift of the NW.

However, based on our investigation, we can state that both the interfaces between the two materials are sharp and there is no As incorporated into the GaP segments of the heterostructure, to the best of our instrumentation sensitivity.

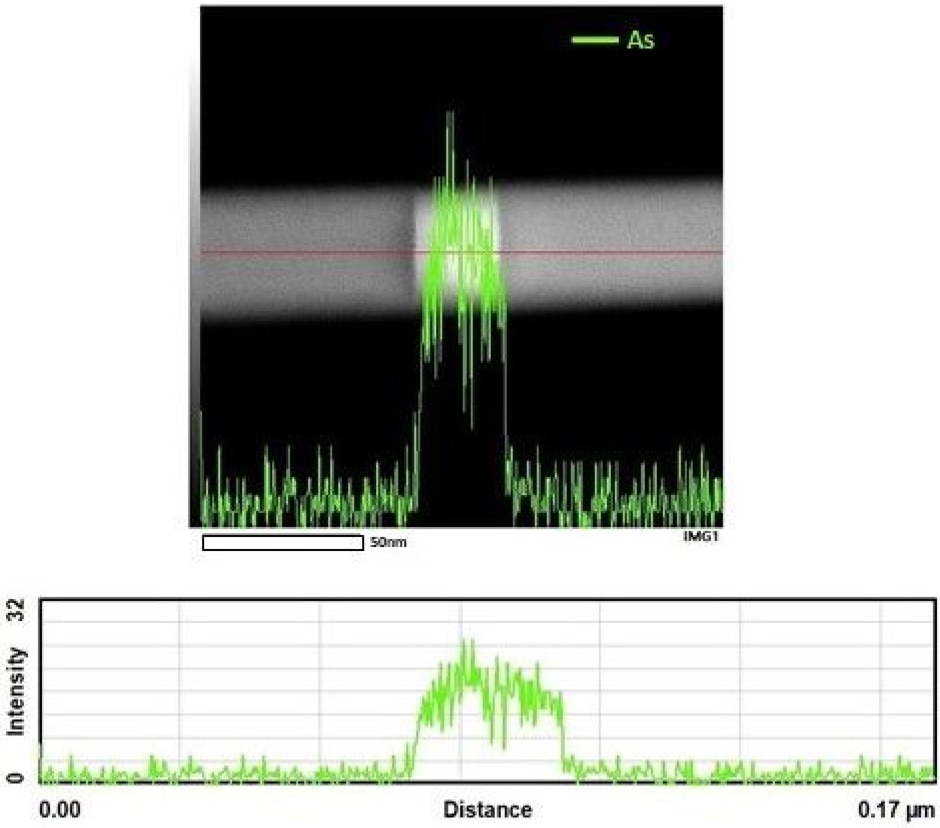

Figure S2: EDX Linescan of a QD with 90% of As grown in a NW with proper tip morphology, superimposed to the STEM image in the top pannel and zoomed in the bottom pannel. The green line is the As intensity along the NW axis. As it can be seen the intensity dropout is extremely sharp, indicating abrupt interfaces between the GaAsP QD and both the upper and lower GaP segments.

S3. GaP Radial Growth

In pursuit of growing a GaP shell around the QD NWs to mitigate the influence of surface electron states on the QD emission and finally obtain thick nanowires that possibly act as waveguides for the QD emission, our investigation focused on understanding the radial and axial growth behavior of GaP as a function of growth temperature and time. We used Au nanoparticles obtained from diluited Au colloidal solution for this purpose.

Having found an optimal temperature for the VLS GaP NW growth, i.e. 560 ± 10°C, we sought to investigate lower growth temperatures to promote radial growth at the expense of axial growth. Moreover, we decided to increase the TEGa and TBP line pressure ratios from 0.4/2.0 Torr to 0. 7/2.5 Torr to enhance the radial growth of the GaP shell around the GaAs/GaP/GaAsP/GaP NW core, as already demonstrated in the main text.

Figure S3 illustrates the axial (panel a) and the radial (panel b) dimensions of the GaP shell as a function of growth time (the last step of the growth procedure illustrated in Figure 1 of the main text, $-\Delta T$ indicating the lower shell growth temperature with respect to the QD and GaP stem growth temperature) observed after a 75 minute growth time at four different temperatures. Notably, radial growth rate exhibits a nearly constant value within the investigated temperatures window, while axial growth decreases with increasing $-\Delta T$ (corresponding to lower temperatures). However, we have found that extremely low temperatures during GaP growth ($-\Delta T \geq 90°C$) induce substantial two-dimensional (2D) growth on the substrate, effectively burying a significant portion of the NW. At the lowest acceptable temperature ($-\Delta T = 70°C$), the NWs showed no measurable optical signals. Transmission electron microscopy (TEM) imaging of such NWs reveals the presence of numerous stacking faults along their whole length (see Figure S4 a), probably explaining the bad optical properties of such NWs. Also, very thick shells obtained at lower $-\Delta T$, i.e. higher growth temperatures, show many stacking faults. On the other hand, NWs with the GaP shell grown at $-\Delta T = 30°C$ and for shorter growth time (30 min) show no defects (see panel b of Figure S4). Figure S5 illustrates the radial (panel a and insets) and axial (panel b) growth of the GaP shell obtained at $-\Delta T$ of 30°C, for different growth times, demonstrating approximately linear trends within the explored time frame. These finding can be used for further optimizing the NW diameter and the quality of the GaP shell to achieve an active NW waveguide, enhancing the photon extraction rate. This aspect will be investigated in a separate paper, while here we limit the optical investigation to the NWs obtained after 30 minutes of GaP shell growth, i.e. with a NW diameter of 100 ± 20 nm which corresponds to a shell thickness of ~30 nm around the QD.

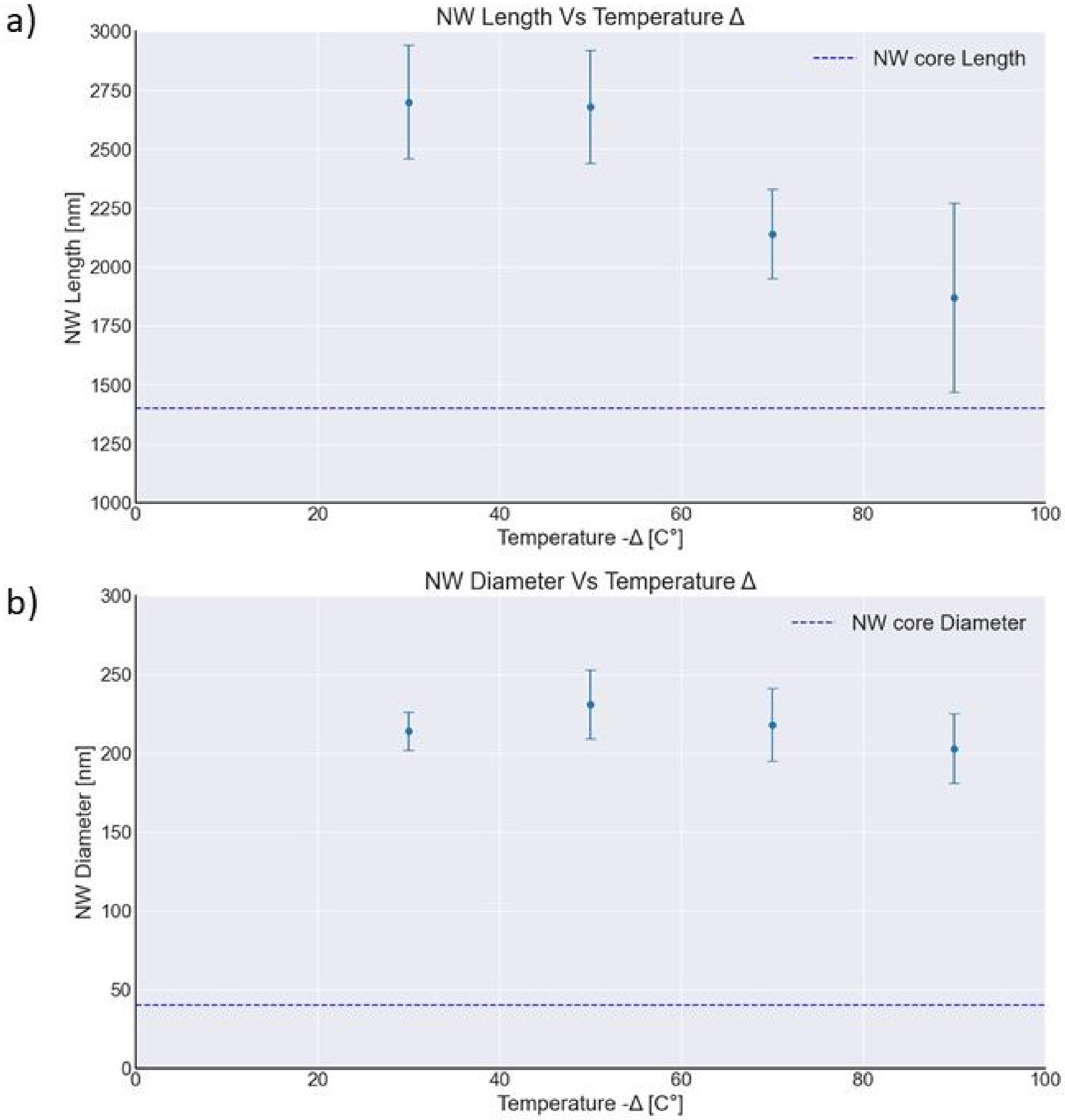

Figure S3: NW length (a) and diameter (b) of the NWs with the GaP shell obtained after 75-minute growth time at four different temperatures (-ΔT with respect to the GaP stem growth temperature). In the lower panel, the diameter has been measured roughly at the height of the QD in the NW. The errors have been derived as the standard deviation of the Gaussian curves from about 100 measurements for each as grown sample. In the upper panel, the length has been measured from the bottom of the NW to the Au nanoparticle, using a 45° tilted SEM image. The error bars have been derived in the same way as above. In both panels, the dotted line represents the GaAs/GaP/GaAsP/GaP heterostructures value for diameter and length, before growing the shell at a lower temperature.

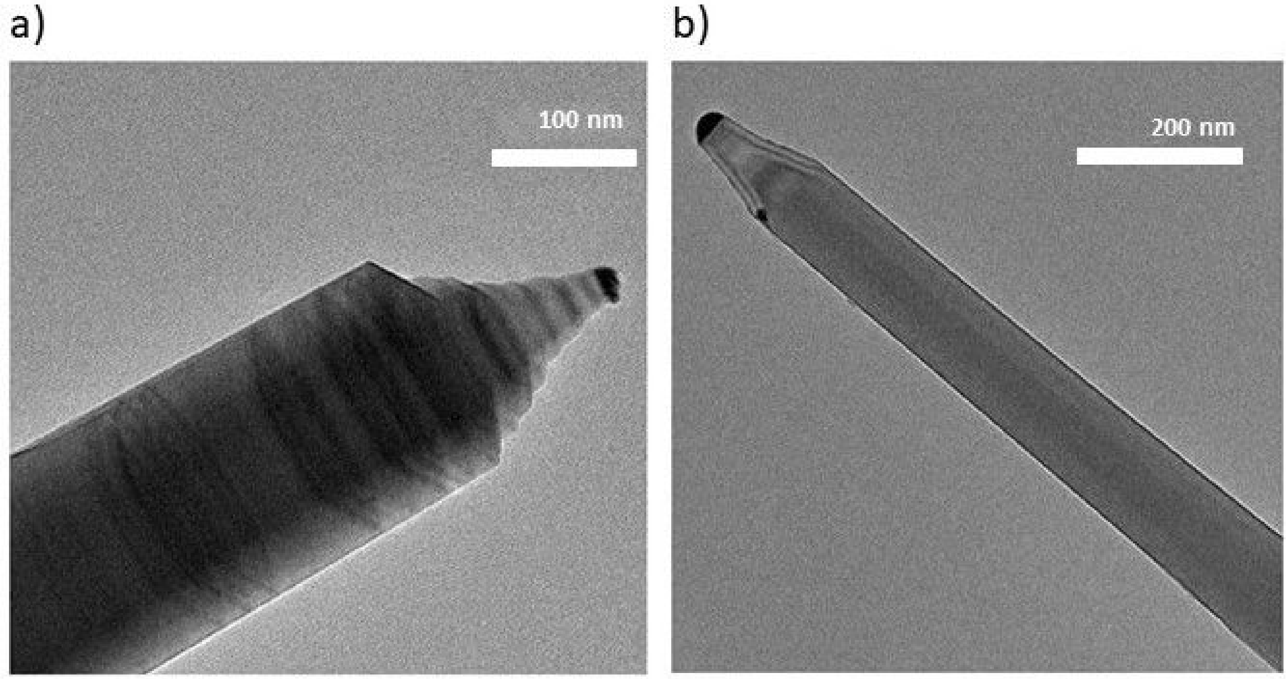

Figure S4: a) Bright-field TEM image of a NW with the shell grown at -ΔT=70°C for 75 min. Stacking faults are visible near the top of the NW. b) Bright-Field TEM image of a NW with the shell grown at -ΔT=30°C for 30 min, where no stacking faults are visible.

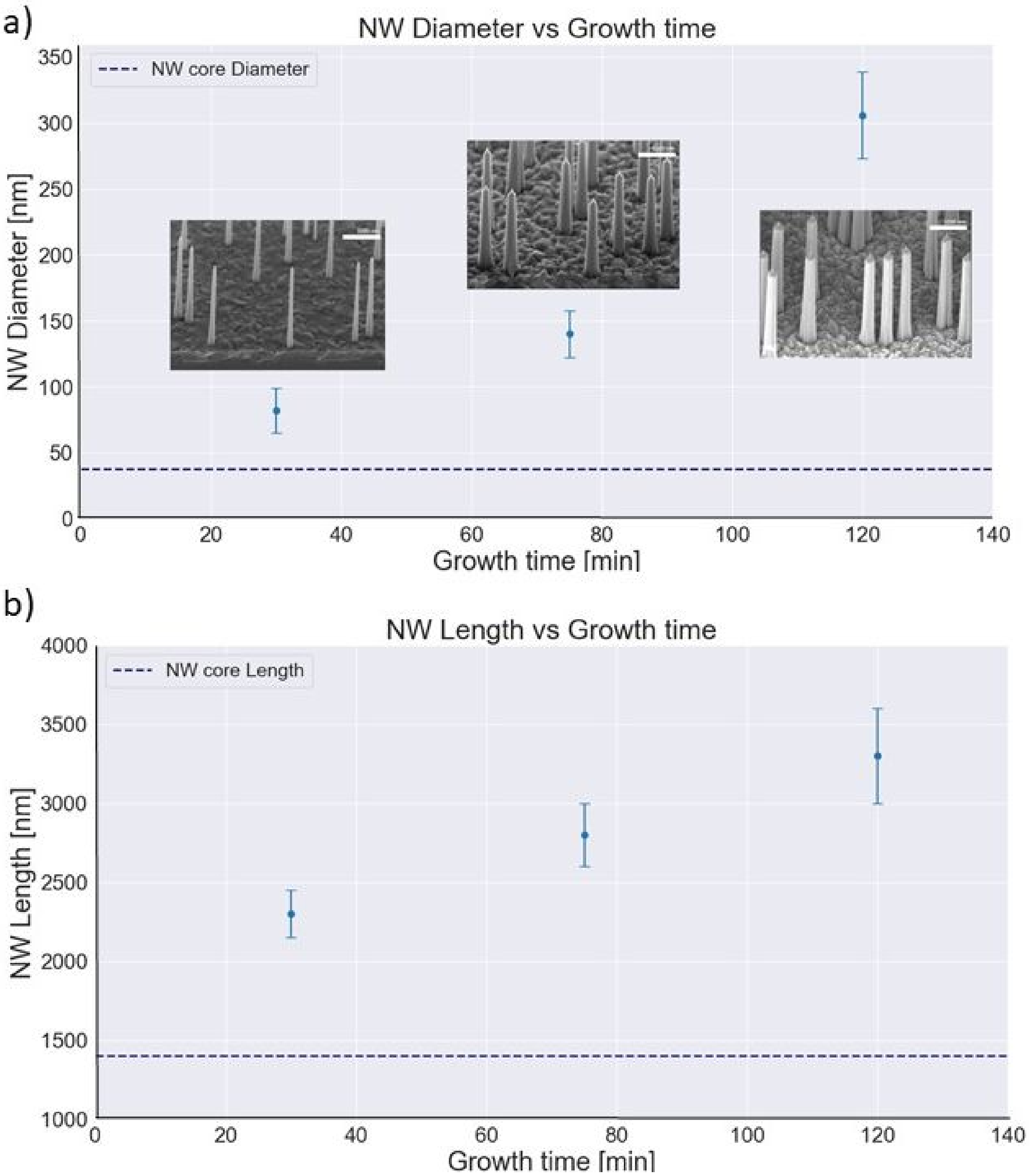

Figure S5: NW diameter (a) and length (b) with the GaP shell realized at -ΔT of 30°C, for different growth times. In the upper panel, the diameter has been measured roughly at the height of the QD in the NW. The errors have been derived as the standard deviation of the Gaussian curves from about 100 NW measurements for each as grown sample. In the lower panel, the length has been measured from the bottom of the NW till just underneath the Au nanoparticle, from a 45°-tilted SEM image. The error bars have been derived in the same way. In both panels, the dotted line represents the shell-less GaAs/GaP/GaAsP/GaP heterostructures value for diameter and length, before growing the shell at a lower temperature. In the insets we show SEM 45° images of the resulting NWs.

S4. Shell Effect

We show in Figure S6 the comparison between micro-photoluminescence (μ-PL) spectra acquired from single QD NWs grown from Au colloids with 70% As content: in blue we show two NWs with a 30 nm-thick GaP shell, grown as explained in section S3; in red we show two NWs nominally without the shell (with just a thin passivation shell, as explained in section 2.3 of the main text).

We observe that the 30nm thick shell does not improve the emission intensity. Instead, the brightness of the QD emission can decrease by up to 40% when compared to the most intense NW without a shell (in Figure S6, NW A - no shell). The luminescence from the QDs of the NWs with 30 nm thick shell (Figure S6) exhibits ~35 meV blue shift of emission energy compared to the QDs emission of the NWs with no shell (with just a few monolayers of GaP as thin passivation shell). This is likely due to the additional compressive strain (in the axial direction) experienced by the As rich (70%) GaAsP QD (large lattice constant) due to the presence of the thicker GaP shell (smaller lattice constant). It is reasonable that some small compressive strain (in all directions) is present also in the NWs with no shell due to the lattice mismatch, but a thicker GaP shell can introduce extra compressive stress on the GaAsP QDs along the NW axis which affects the QDs band structure and introduce blue shift compared to no shell QD NWs. In WZ GaAs, a compressive strain blueshifts the PL peak, so our results on WZ GaAsP are in good qualitative agreement with that observation[1].

In the spectra, we also observe that the presence of the GaP shell is responsible for the appearance of an additional weak and broad emission band, spanning from 1.7 eV to 2.2 eV. As we will see in the next section, we have attributed this signal background to the impurities incorporated into GaP and defect states originating during the growth, that are preferentially incorporated at low temperatures, i.e. during the thick shell growth. These results suggest that the QD surface passivation already occurs in NWs nominally without the shell, owing to the thin (few monolayers) passivation layer, while a thicker shell grown at low temperature does not improve the QD emission. This would probably happen if the shell quality was improved and the thickness optimized to match the optimal NW diameter to an efficient waveguide for the emitted photons[2] . Moreover, the tapering length and angle of the final NW should also be properly tailored to increase the convergence of the emitted light and increase the photon extraction efficiency[2]. This can explain our results and suggest that an improvement of the QD emission intensity can be reached by further optimizing the final NW geometry in the future.

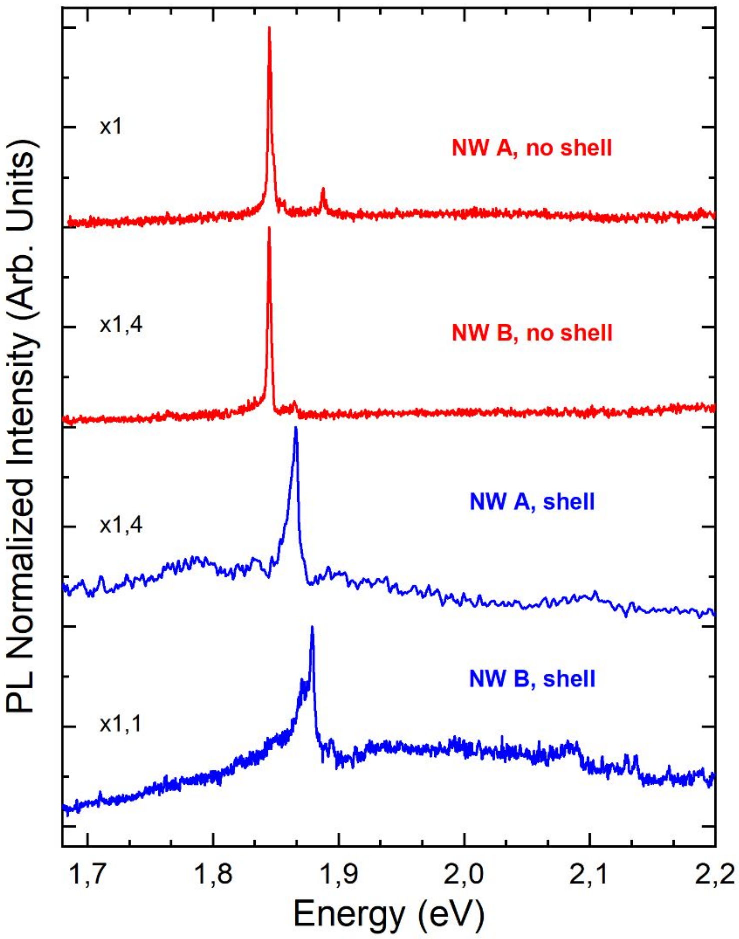

Figure S6: μ-PL spectra comparison of single GaAsP QD NWs (70% As content) with (blue) and without (red) 30nm thick shell. T = 5.5K, excitation power 200 nW. NW A and B show representative spectra from two different single NWs. The 30nm-thick shell of GaP does not improve emission intensity, as shown by the normalization factors, but introduces instead an underlying weak broad band.

S5. GaP Emission

In this section, we analyze the emission spectrum of the reference sample of pure GaP NWs, i.e. without QDs, and identify its contributions to later distinguish them from the GaAsP spectrum analysis. In Figure S7, we show μ-PL spectra acquired on an ensemble of GaP NWs at 5.5K. μ-PL from different points of the sample (spectra a-d) shows similar characteristics: an intense band centred at 1.893 eV and FWHM of 0.5 eV (vertical dashed line), that we attribute to incorporation of a large concentration of impurities[3], which in our case can be identified as C, that is always present in growth systems with metalorganic precursors like our CBE, and Sb traces, due to the growth of antimonides in the same chamber.

At energies between 2.05 eV and 2.2 eV (see orange dashed rectangle), the spectra exhibit narrow lines that originate from recombination of shallower bound exciton states with donor/acceptor impurity atoms (again C and Sb are the most probable), with small variations in peak energy and intensity between each measured point. At 2.279 eV and 2.284 eV (in spectra (c) and (d) of Figure S7, red dashed circle), it is possible to observe, respectively, the LO and TO phonon modes of the WZ phase of GaP, in agreement with what was previously calculated and measured[4].

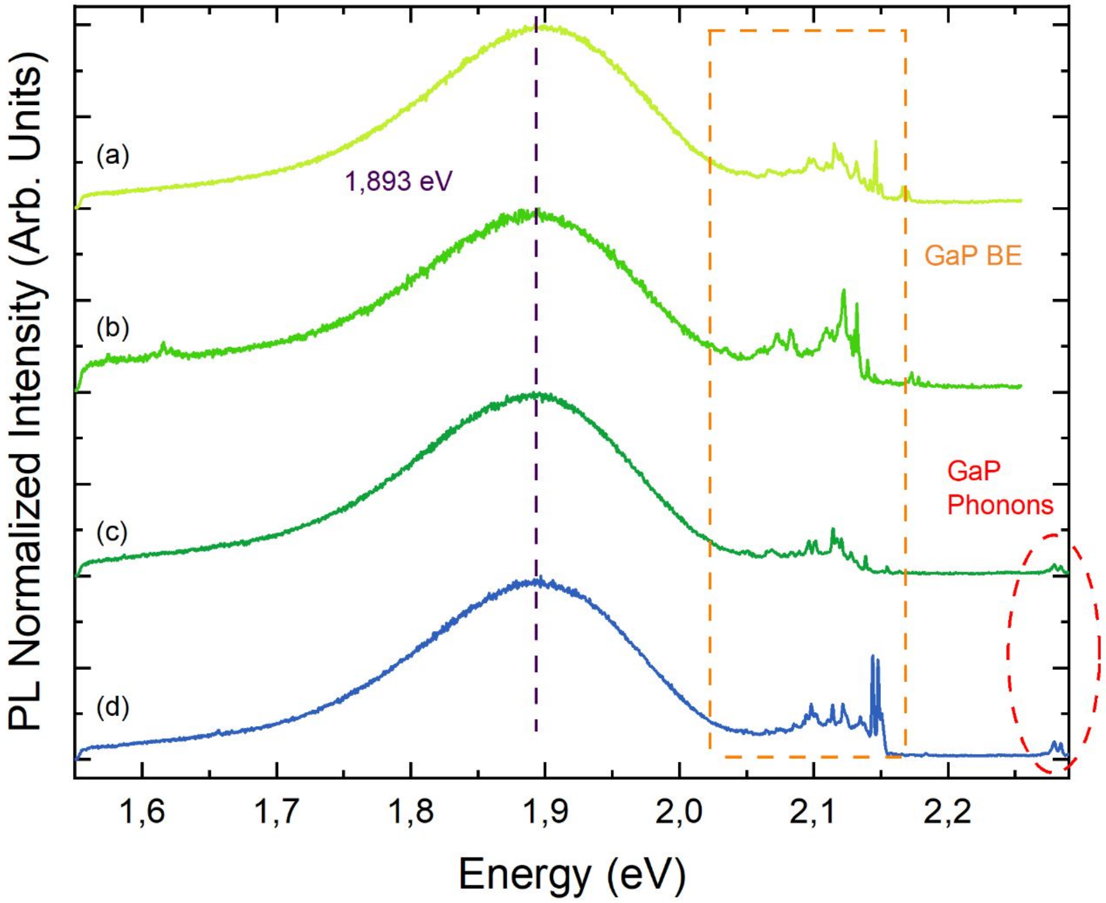

Figure S7: μ-PL spectra taken at T = 5.5K with an excitation power of 500 nW, on different points (a, b, c, d) of an ensemble of GaP NWs (no QD). The orange rectangle highlights the GaP bound exciton region, and the red circle shows the WZ GaP phononic modes LO and TO, see spectra (c) and (d).

Bound Excitons and Phonon Replicas: To gain further insights into the narrow emission lines between 2.05 eV and 2.2 eV of the GaP emission spectra, Figure S8 shows a representative spectrum zoomed on that spectral region. We identify the most intense contributions as excitons bound to impurities (BE), and we label the peak at 2.146 eV as α, and the peaks at 2.166 eV and 2.170 eV as β_1 and β_2 respectively. α and β_1 were previously observed in WZ GaP NWs[5]. These lines are characterized by a FWHM of 1.52 meV, 1.75 meV and 1.42 meV respectively. At lower energies, we see a distribution of less intense peaks which from comparison with theory and experiments[4,5] are identified as phonon replicas related to excitonic peaks. Table S1 shows the expected shifts (ΔE) from the excitonic peaks due to the replicas of the TA (ΔE = 14 meV) acoustic phonon mode, and the TO (ΔE = 45 meV) and LO (ΔE = 50 meV) optical phonon modes, as also indicated by labels in the figure. Single NWs show similar but much weaker emission spectra.

**Phonon Mode**	**ΔE (meV)**	**Energy (eV) - α**	**Energy (eV) - β_1**	**Energy (eV) - β_2**
TA	14	2.132	2.152	2.156
2TA	27	2.119	2.139	2.143
TO	45	2.101	2.121	2.125
LO	50	2.096	2.166	2.120
TO+TA	59	2.087	2.107	2.111
LO+TA	64	2.082	2.102	2.106
2TO	90	2.056	2.076	2.080
2LO	99	2.047	2.067	2.071

Table S1: List of phonon replicas observed in Figure S8. The second column reports the expected shift from the excitonic peaks due to the indicated phonon replicas. Columns three to five show the energy of each phonon replica peak related to the three BEs.

Power-dependent measurements on peaks α, β_1, and β_2 show no red-shift and high saturation power. We report the slope of the integrated intensity of PL as a function of the power fitted with power law: $I \propto (P_{exc})^M$ (Eq. 1 of the main text). We estimate $M_\alpha = (1.007 \pm 0.001)$. The linear scaling of the intensity with excitation power is characteristic of single excitonic recombination. For the β_1 and β_2, we report slopes of $M_{\beta 1} = (1.0 \pm 0.4)$ and $M_{\beta 2} = (1.33 \pm 0.07)$, respectively. The trend of the two β peaks is compatible with charged excitons formed due to inhomogeneous charge capture within the NW structure.

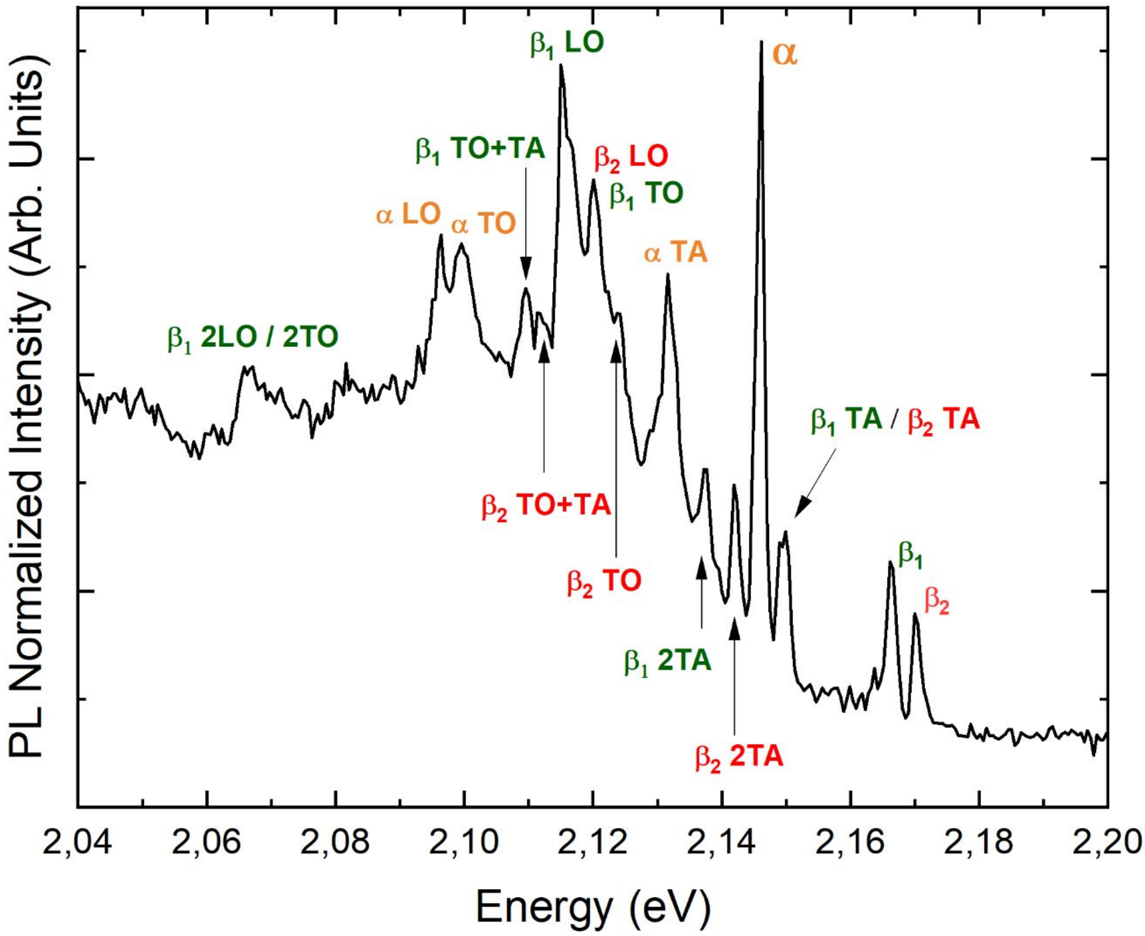

Figure S8: μ-PL spectrum on a single point of GaP NW ensemble, measured at T = 5.5K with an excitation power of 200 nW. Zoom on the narrow excitonic lines spectral region. Phonon replicas peaks related to each exciton are highlighted with different colors.

We performed temperature-dependent μ-PL measurements on the GaP NWs ensemble (Figure S9 panels a and b). We observe a fast quenching of the narrow emission peaks while the broad impurity band gains intensity without reaching saturation and dominates the emission above 75K. This indicates that the radiative recombination of the narrow emission lines originates from shallower impurities (binding energy < 5 meV) than those composing the band at 1.89 eV.

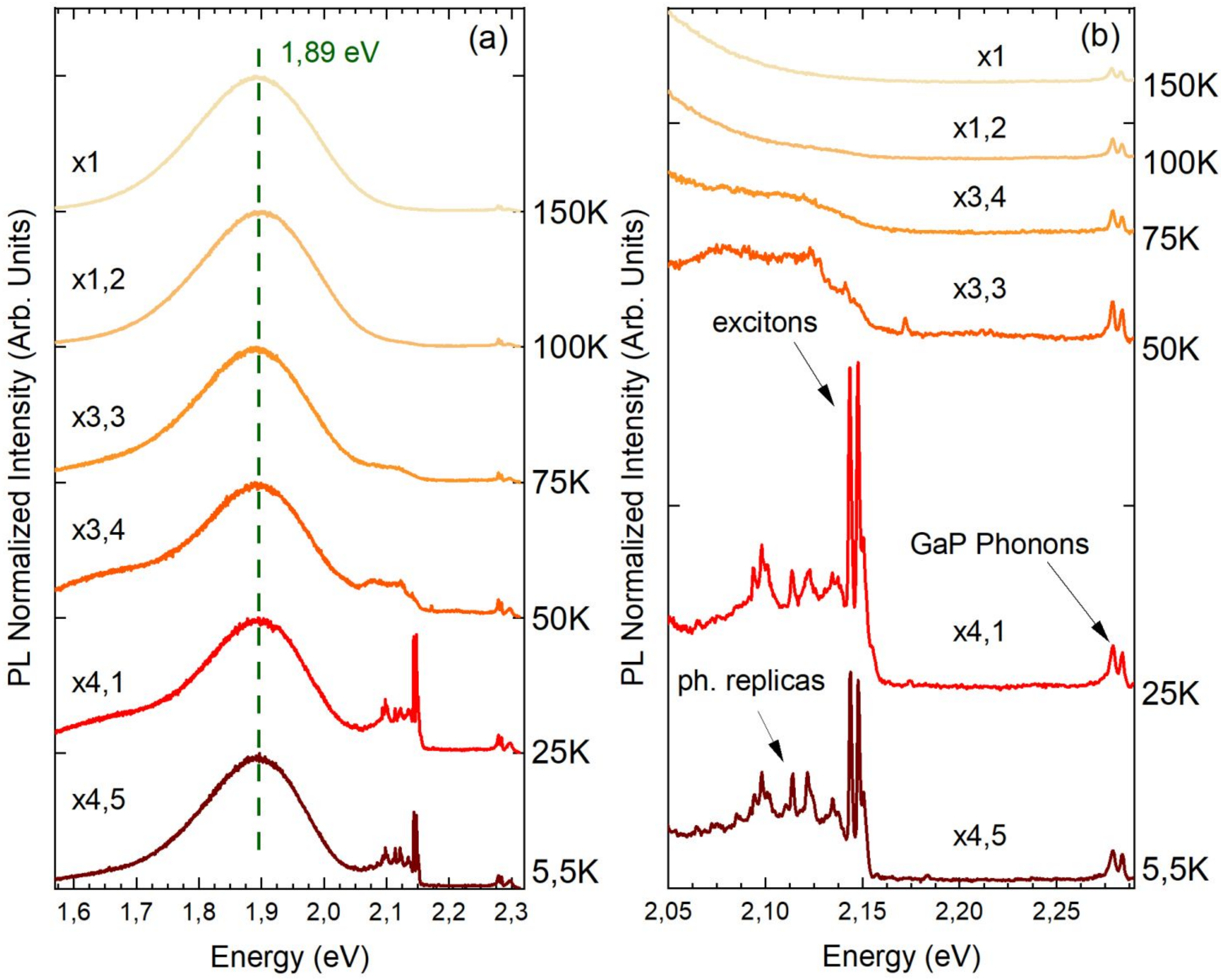

Figure S9: Temperature-dependent μ-PL spectra on GaP NW ensemble. (a) The broad emission band from impurities (green dashed line) gains intensity, as highlighted by the normalization factors on the left side. (b) Zoom on the BE spectral region, showing quenching of the narrow excitonic emissions and pertinent phonon replicas. At 50K the impurity states do not exhibit narrow recombination peaks anymore. Only the GaP phonons remain visible.

S6. GaAsP QD NWs – As = 50%

We have identified the characteristic emission of GaP in the S5, we can now compare it with μ-PL spectra on samples containing QD NWs with 50% As content. These samples do not exhibit any narrow peak attributable to QD emission. The spectra are dominated by the broad band of impurities, see Figure S10, and none of the measured NWs allow us to identify GaAsP 50% emission band/peak. The absence of a QD-like peak in this sample can be attributed to an unfavorable band alignment:

preliminary calculation performed in the zincblende phase predicts an energy separation between GaP and QD bandgap of 50 meV, which may not be enough to create a favorable recombination center for photogenerated carriers, which instead recombine in the GaP barriers. A smaller QD could instead promote a larger quantum confinement.

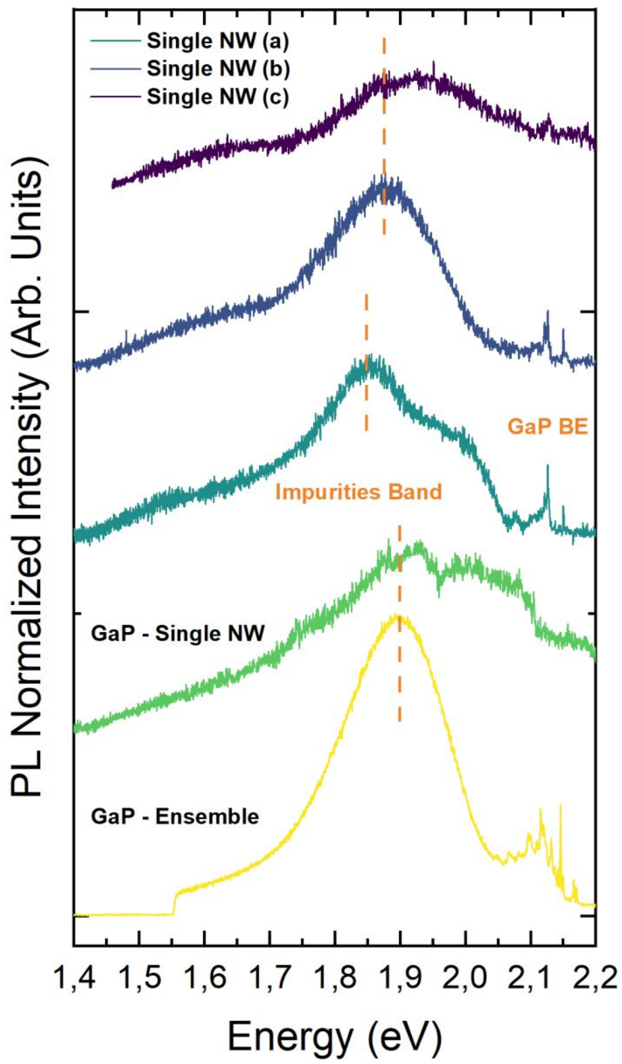

Figure S10: μ-PL spectra, at 5.5K, of single GaAsP QD NWs (As = 50%) compared to spectra of a single NW and an ensemble of NWs of GaP. The orange dashed lines mark the main impurities band, visible in both NWs with and without QD. The above discussed excitonic narrow lines are also visible.

S7. Additional Optical Spectroscopy Data

In this section, we present additional optical measurements on a single QD NW with 90% As content with a 30nm thick shell. In Figure S11, a μ-PL map is displayed, acquired by moving along the NW long axis with 400nm steps. The spectra in panel (a) show a broad emission composed of a high-intensity peak, at 1.754 eV, localized in a region of 800 nm which we attribute to the QD. The QD emission energy is shifted according to its As content: higher As content leads to lower emission energy, as expected and discussed in Figure 4 in the main text. The peak at 1.776 eV, could instead be related to radiative recombination in the p-shell of the QD. The QD main peak exhibits a FWHM of 10 meV which is relatively higher compared to the average value of measured QD FWHM of (5.2 ± 1.6) meV, suggesting a low degree of confinement for carriers that results in the discrete energy levels to be closer in energy than in the QD with 70% As content, and explain why the p-shell is closer in energy.

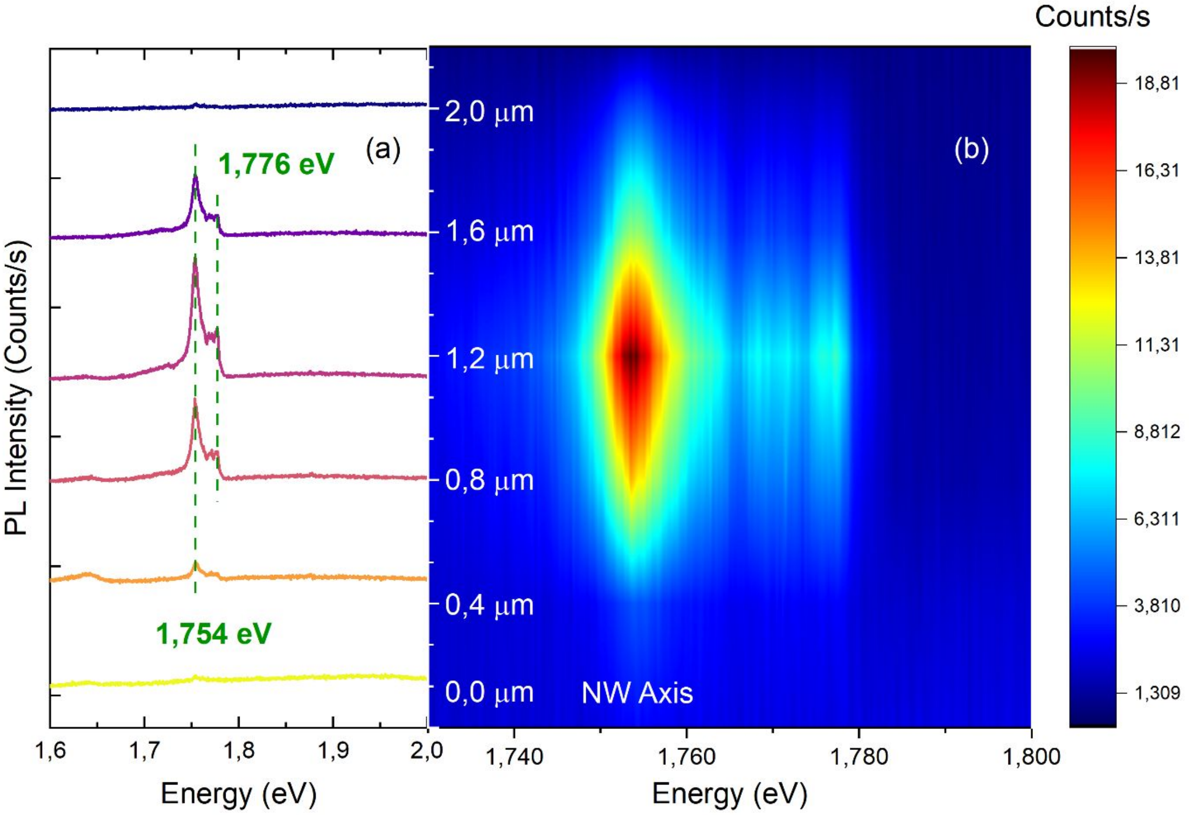

Figure S11: μ-PL spectra of a single GaAsP QD NW with As = 90%, T = 5.5K at excitation power of 1μW (a) μ-PL map along the NW axis. QD emission at 1.754 eV with FWHM of 10 meV. (b) Color plot of the intensity of the emission along the NW axis, zoom on the QD emission spectral range. Single spectra singled out by this plot are shown in panel (a) on a wider spectral range.

Measurements show the locality of the emitter, whose intensity is localized in an 800 nm long zone, comparable with our laser spot size.

Nevertheless, as can be seen in panel (b) of Figure S11, also for As content equal to 90%, we have created a favorable recombination center for photogenerated carriers, even if they're created in the GaP barriers where most of the light is absorbed.

Figure S12 a), shows spectra as a function of excitation power on the same QD NW. Similar to what was observed for the NW with 70% As content (in the main text, section 2.5.3), we see a peak with energy higher by 5 meV than the QD emission dominating the spectrum at low powers and then reaching saturation at 200 nW (0.2 P_0). At higher excitation powers, the QD emission, at 1.754 eV (green dashed line), gains in intensity and becomes the main contribution to the emission. In Figure S12 b), we have fitted the integrated intensity of the QD peak with the power law $I_{exc} \propto (P_{exc})^M$ (Eq. 1 of the main text) which yields a slope of M_X = 1.16 ± 0.26, suggesting a single excitonic recombination process (X). This assumption is further confirmed by the invariance of the emission energy as a function of the excitation power and saturation of intensity at high excitation power. At $2P_0$ the peak at 1.768 eV (red dashed line) starts gaining intensity. The integrated PL intensity dependency with power once fitted with the power law results in a coefficient of M_{X*} = 1.3 ± 0.1, compatible with a charged exciton recombination process. The spectra also show that the peak at 1.776 eV (blue dashed line) gains intensity as the excitation power increases, without showing any red-shift. We estimated a power law coefficient of 1.0 ± 0.17. The emission could likely be related to excitonic recombination in the p-shell of the QD.

Figure S12 c) shows μ-PL spectra as a function of temperature. The study was conducted on the same NW of the power study. Similar to what is reported in the main text for the QD NW with 70% of As, we observe a rapid decrease in the intensity of the QD peak with the increasing temperature, as highlighted by the normalization factors. The QD emission undergoes "Varshni"-like red-shift: 27 meV at 150K. As the temperature increases we observe that the higher energy states start to populate until the two peaks, at 1.754 eV (green dashed line) and 1.776 eV (blue dashed line), show the same intensity. The "Arrhenius" plot of the integrated intensity of the QD peak at 1.754 eV is shown in panel (d). The data are fitted considering two active non-radiative recombination channels for the exciton, according to the equation:

$$I = \frac{1}{1 + c_1 * \exp\left(-\frac{E_a}{k_B T}\right) + c_2 * \exp(-\frac{E_b}{k_B T})} \quad Eq.S1$$

Where c_1 and c_2 are defined as the ratio between the lifetime of the exciton and the time it takes to be captured by a non-radiative recombination center. E_a and E_b are the activation energies and k_B is the Boltzmann constant. We estimate $E_a = (14 \pm 2)$ meV and $E_b = (3 \pm 3)$ meV. E_b is likely due to a loosely bound exciton, hidden within the large FWHM and soon ionized with temperature below 30K, while E_a corresponds to activation of a second recombination channel (most likely the peak at 1.776 eV), thus carriers escape to this excited state that broadens and merge to the X peak when the temperature is above 150K.

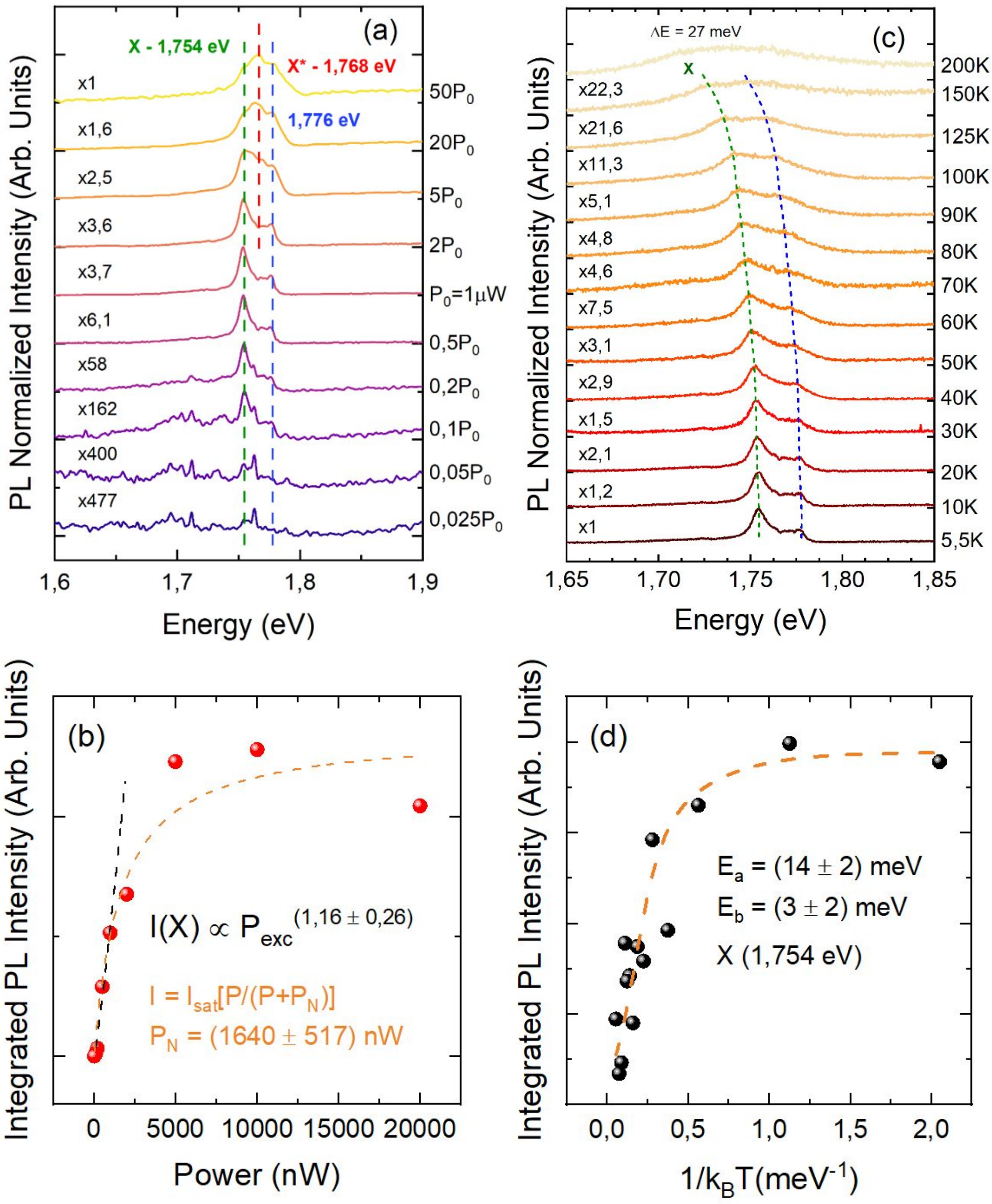

Figure S12: Power and temperature dependent μ-PL measurements on single GaAsP QD NWs with As = 90%. (a) Power-dependent spectra of the QD peak acquired at 5.5K, with intensity normalization factors. $P_0 = 1\mu W$. The green dashed line highlights the single exciton (X) emission peak. The red dashed line marks the charged exciton peak (X*). The blue dashed line shows the peak at 1.776 eV. (b) The plot of the integrated intensity (obtained from the spectra in panel (a)) of peak X as a function of power. The dashed orange line shows a fit with $I = I_{sat}[\frac{P}{P+P_N}]$ (Eq. 2 of the main text) where P_N is the laser power at which the intensity is half of $I_{sat.}$ The black dashed line represents the Power law fit that yields the single exciton behavior of X. (c) μ-PL temperature-dependent spectra, acquired at the power of 1μW. The green and the blue dashed lines show the thermal red-shift of the X peak and the peak at 1.776 eV, respectively. (d) Integrated PL intensity of the X peak as a function of the reciprocal of the temperature, with Arrhenius fit (orange dashed line) and extracted activation energy.

S8. Calculation of the Bohr Exciton Radius

We performed calculations to estimate the Bohr exciton radius (EBR) in WZ GaAsP, since quantum confinement effects appear as the confining dimensions approach the e-h distance. We interpolated according to the As% starting from the EBR of WZ GaP and WZ GaAs using the carrier masses and dielectric constants estimated in the theoretical works in[6,7]. In the WZ crystal symmetry, the carrier will experience different masses [6,7,8] (and refractive indexes) when moving parallel or perpendicular to the WZ c-axis, and so two values of the hole and electron masses (and refractive indexes) are calculated and considered as weighted average (twice the contribution of motion perpendicular to the WZ c-axis and once the contribution parallel to it).

Our calculations result in an EBR of 2.30 nm for GaP and 2.98 nm for GaAs. The GaAs value is significantly lower than the EBR in the ZB phase, 13 nm, due to the increased carriers' mass in WZ. This is quite puzzling, given that in WZ GaAs NWs with 10 nm diameter quantum confinement was clearly observed[9], and it might suggest that theoretically calculated masses are slightly different than real ones. Using these values, our calculations yield an EBR of 2.9 nm for dots with 90% As and 2.8 nm for 70% As, which are very small values compared to all our QD sizes. Ref.[9] also estimated the carriers' masses, although not specifying their direction. If we use their GaAs values ($0.1m_0$ for electron and $0.5m_0$ for holes, without averaging on carriers' motion direction) and the theoretical GaP values from ref.[10], we get an EBR for GaAs of 12.9 nm and for GaAsP of 9.7 nm and of 11.8 nm for As% of 70 and 90 respectively. These values, if correct, would be still smaller than our QD diameter (~ 30 nm) but more comparable to it, which may suggest that we (and the other works in the field mentioned in the introduction) have grown a weakly confined QD.

References

(1) Signorello, G.; Lörtscher, E.; Khomyakov, P. A.; Karg, S.; Dheeraj, D. L.; Gotsmann, B.; Weman, H.; Riel, H. Inducing a Direct-to-Pseudodirect Bandgap Transition in Wurtzite GaAs Nanowires with Uniaxial Stress. *Nat Commun* **2014**, *5* (1), 3655.

(2) Mäntynen, H.; Anttu, N.; Sun, Z.; Lipsanen, H. Single-Photon Sources with Quantum Dots in III–V Nanowires. *Nanophotonics* **2019**, *8* (5), 747–769.

(3) da Silva, B. C.; Couto Jr, O. D. D.; Obata, H.; Senna, C. A.; Archanjo, B. S.; Iikawa, F.; Cotta, M. A. Wurtzite Gallium Phosphide via Chemical Beam Epitaxy: Impurity-Related Luminescence vs Growth Conditions. *ACS Omega* **2022**, *7* (48), 44199–44206.

(4) De Luca, M.; Fasolato, C.; Verheijen, M. A.; Ren, Y.; Swinkels, M. Y.; Kölling, S.; Bakkers, E. P. A. M.; Rurali, R.; Cartoixà, X.; Zardo, I. Phonon Engineering in Twinning Superlattice Nanowires. *Nano Lett* **2019**, *19* (7), 4702–4711.

(5) Assali, S.; Greil, J.; Zardo, I.; Belabbes, A.; De Moor, M. W. A.; Koelling, S.; Koenraad, P. M.; Bechstedt, F.; Bakkers, E. P. A. M.; Haverkort, J. E. M. Optical Study of the Band Structure of Wurtzite GaP Nanowires. *J Appl Phys* **2016**, *120* (4).

(6) De, A.; Pryor, C. E. Predicted Band Structures of III-V Semiconductors in the Wurtzite Phase. *Physical Review B—Condensed Matter and Materials Physics* **2010**, *81* (15), 155210.

(7) De, A.; Pryor, C. E. Optical Dielectric Functions of III-V Semiconductors in Wurtzite Phase. *arXiv preprint arXiv:1011.3081* **2010**.

(8) Tedeschi, D.; Fonseka, H. A.; Blundo, E.; Granados del Águila, A.; Guo, Y.; Tan, H. H.; Christianen, P. C. M.; Jagadish, C.; Polimeni, A.; De Luca, M. Hole and Electron Effective Masses in Single InP Nanowires with a Wurtzite-Zincblende Homojunction. *ACS Nano* **2020**, *14* (9), 11613–11622.

(9) Vainorius, N.; Lehmann, S.; Gustafsson, A.; Samuelson, L.; Dick, K. A.; Pistol, M.-E. Wurtzite GaAs Quantum Wires: One-Dimensional Subband Formation. *Nano Lett* **2016**, *16* (4), 2774–2780.

(10) Pelant, I.; Valenta, J. *Luminescence Spectroscopy of Semiconductors*; OUP Oxford, 2012.
